# Nanocomposite Concept for Electrochemical *In Situ* Preparation of Pt–Au Alloy Nanoparticles for Formic Acid Oxidation

**DOI:** 10.1021/jacsau.2c00335

**Published:** 2022-07-06

**Authors:** Jia Du, Jonathan Quinson, Damin Zhang, Baiyu Wang, Gustav K. H. Wiberg, Rebecca K. Pittkowski, Johanna Schröder, Søren B. Simonsen, Jacob J. K. Kirkensgaard, Yao Li, Sven Reichenberger, Stephan Barcikowski, Kirsten M. Ø. Jensen, Matthias Arenz

**Affiliations:** † Department of Chemistry, Biochemistry and Pharmaceutical Sciences, University of Bern, 3012 Bern, Switzerland; ‡ Department of Chemistry, University of Copenhagen, 2100 Copenhagen, Denmark; § Department of Biochemical and Chemical Engineering, University of Aarhus, 8200 Aarhus, Denmark; ∥ Department of Energy Conversion and Storage, Technical University of Denmark, 2800 Lyngby, Denmark; ⊥ Department of Food Science, University of Copenhagen, 1958 Frederiksberg, Denmark; # Niels-Bohr-Institute, University of Copenhagen, 2100 Copenhagen, Denmark; ¶ Technical Chemistry I and Center of Nanointegration Duisburg Essen (CENIDE), University of Duisburg-Essen, 45141 Essen, Germany

**Keywords:** nanocomposite electrocatalysts, formic acid oxidation reaction, gas diffusion electrode setup, small-angle X-ray scattering, in situ alloying

## Abstract

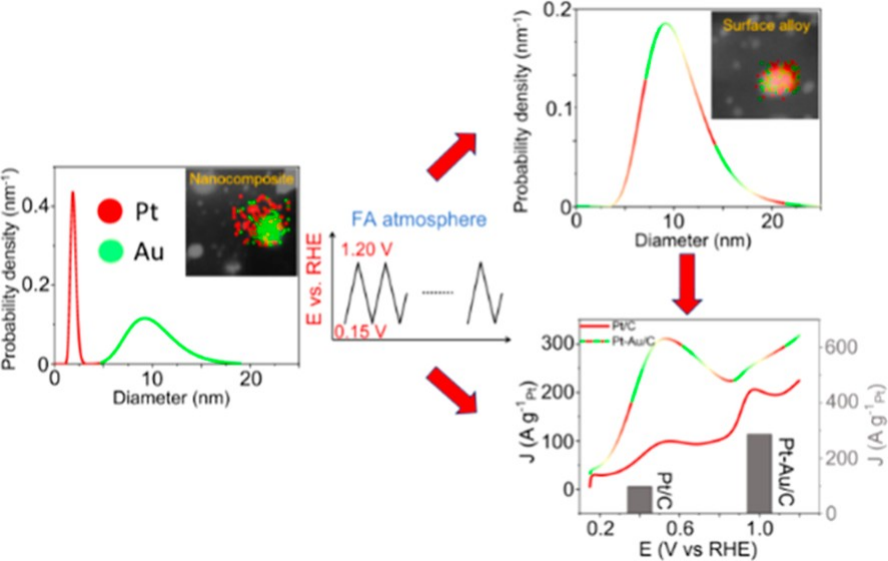

Herein, we report a straightforward approach for the *in situ* preparation of Pt–Au alloy nanoparticles from Pt + *x*Au/C nanocomposites using monometallic colloidal nanoparticles as starting blocks. Four different compositions with fixed Pt content and varying Pt to Au mass ratios from 1:1 up to 1:7 were prepared as formic acid oxidation reaction (FAOR) catalysts. The study was carried out in a gas diffusion electrode (GDE) setup. It is shown that the presence of Au in the nanocomposites substantially improves the FAOR activity with respect to pure Pt/C, which serves as a reference. The nanocomposite with a mass ratio of 1:5 between Pt and Au displays the best performance during potentiodynamic tests, with the electro-oxidation rates, overpotential, and poisoning resistance being improved simultaneously. By comparison, too low or too high Au contributions in the nanocomposites lead to an unbalanced performance in the FAOR. The combination of *operando* small-angle X-ray scattering (SAXS), scanning transmission electron microscopy (STEM) elemental mapping, and wide-angle X-ray scattering (WAXS) reveals that for the nanocomposite with a 1:5 mass ratio, a conversion between Pt and Au from separate nanoparticles to alloy nanoparticles occurs during continuous potential cycling in formic acid. By comparison, the nanocomposites with lower Au contents, for example, 1:2, exhibit less *in situ* alloying, and the concomitant performance improvement is less pronounced. On applying identical location transmission electron microscopy (IL-TEM), it is revealed that the *in situ* alloying is due to Pt dissolution and re-deposition onto Au as well as Pt migration and coalescence with Au nanoparticles.

## 1 Introduction

Fuel cells, electrochemical devices that convert the chemical energy of a fuel into electrical energy, are considered the most promising candidates for powering not only large automobiles and heavy-duty electric trucks^1−3^ but also smaller devices.^4^ Therefore, fuel cells attract considerable attention in both industrial and academic fields.^2,5^ The most common fuels are classified as gaseous, for example, hydrogen, and liquid, for example, ethanol, methanol, and formic acid (FA). Hydrogen-fed proton exchange membrane fuel cells (PEMFCs) have been studied intensively.^6,7^ Well-known for their high-power density, they have been, for instance, applied in cars, buses, and trucks. However, intrinsic restrictions such as a lack of a widespread hydrogen distribution network still exist.^6,7^ Alternative fuel cells using liquid fuels are the so-called direct methanol, ethanol, and formic acid fuel cells (DMFCs, DEFCs, and DFAFCs). Compared with DMFCs and DEFCs, DFAFCs exhibit a lower crossover of FA through the polymer membrane^8^ and a smaller onset overpotential^9^ which makes DFAFCs promising as a power source for portable electronics.^10^

Pt is one of the most commonly used electrocatalyst material in FAOR studies. However, Pt is prone to be poisoned by CO_ad_, which in literature is linked to a dehydration or disproportionation pathway of FAO and which reduces the efficiency of a fuel cell.^11,12^ To alleviate poisoning and to improve the FAO electrocatalytic activity, combining Pt with a foreign metal to form Pt-based bimetallic catalysts is regarded as an effective strategy.^13−15^ Pt-M bimetallic (M = Bi, Pd, Au, *etc.*) catalysts have been developed and display improvements in FAOR catalytic performance.^16,17^ Among the different bimetallic Pt–M catalysts, Pt–Au exhibits a high resistance of Au dissolution in acidic media^18,19^ as well as an improved FAO activity.^20,21^ Various structures of Pt–Au bimetallic materials, such as PtAu alloys,^22,23^ PtAu core–shell particles,^24,25^ Pt deposited onto Au,^26,27^ and so forth have been synthesized. The observed improved performance is ascribed to either a modified electronic structure^28,29^ or an ensemble effect.^30,31^ Organic surfactants are typically needed to form a specific nanostructure. These additives are likely to block surface sites of the active phase. Removing surfactants from nanomaterials typically requires a post-cleaning step to the catalyst surface that may cause adverse effects on the catalytic performance.^30^ However, Guay and co-authors reported a laser ablation method to prepare surfactant-free Pt–Au bimetallic catalysts, avoiding a surfactant-removal process, and the obtained Pt–Au mixed and Pt–Au alloyed nanoparticles are used for FAO study.^32^

In the present study, we use our previously introduced nanocomposite concept^33,34^ to prepare Pt + *x*Au/C bimetallic nanocomposites with tunable Pt to Au ratio. Surfactant-free, metallic Pt and Au nanoparticles serve as starting blocks. Pt/C serves as a reference catalyst. The FAOR is probed for the different electrocatalysts in a gas diffusion electrode (GDE) setup, a newly developed fuel cell catalyst testing platform that was recently used to benchmark the commercial Pt/C and Pd/C for electro-oxidation of small organic compounds.^35^*Operando* small-angle X-ray scattering (SAXS) is combined to monitor the particle size distribution during FAOR potentiodynamic tests. The results indicate that electrochemical potentiodynamic conditions lead to *in situ* alloy formation of the nanocomposite with initially separate Pt and Au nanoparticles. The formed alloyed nanoparticles lead to a substantial improvement in FAO performance compared with the reference catalyst.

## 2 Results and Discussion

### 2.1 Characterization of the as-prepared Electrocatalysts

**Figure 1:**
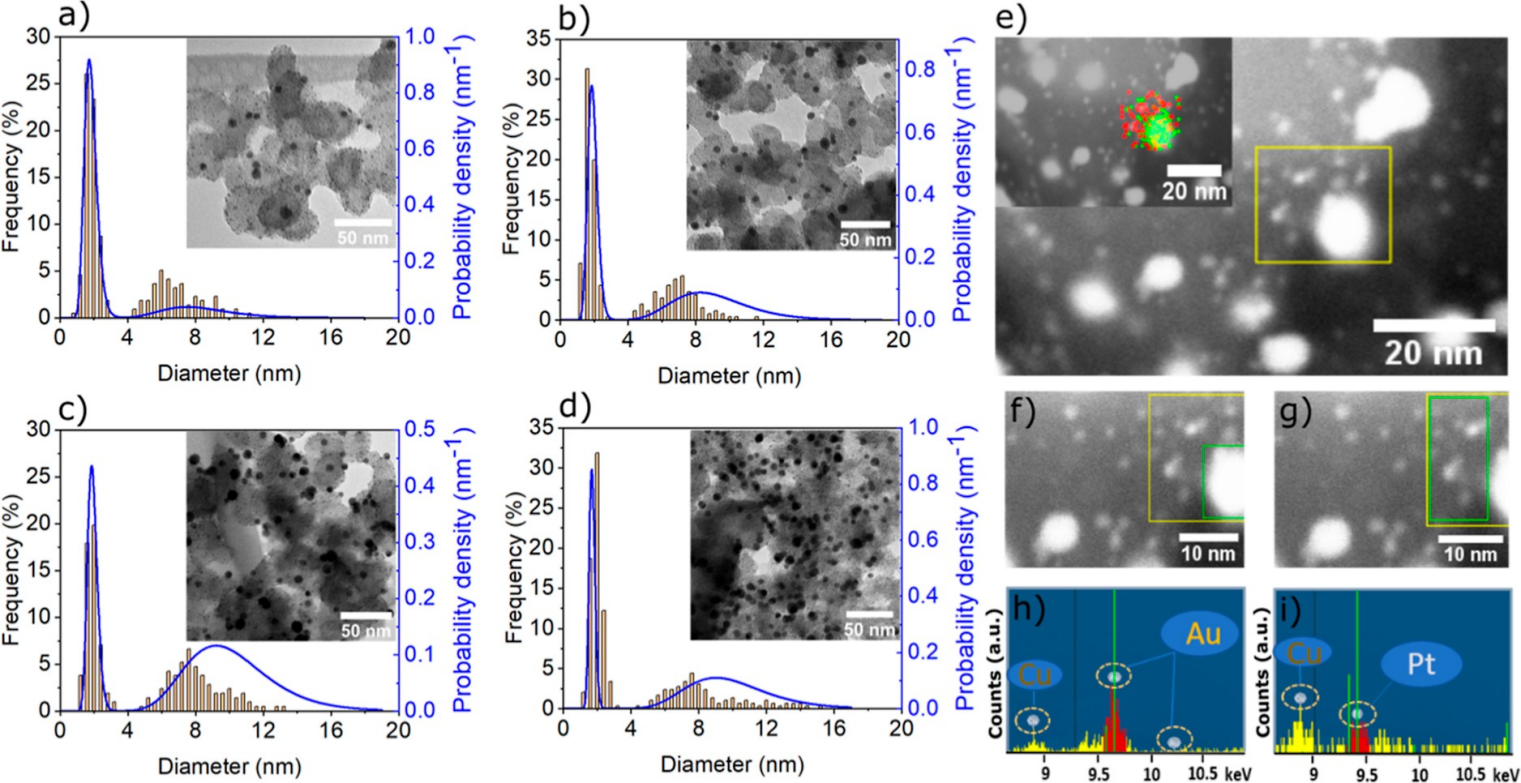
TEM micrographs and size distributions retrieved from TEM (bar diagrams, left axis) and SAXS (blue curves, right axis) of pristine Pt + Au/C (a), Pt + 3Au/C (b), Pt + 5 Au/C (c), and Pt + 7Au/C (d). STEM micrographs of Pt + 5Au/C with inserted EDX elemental map of Pt (red) and Au (green) (e). STEM micrographs of the selected large bright spot (green square) from Pt + 5Au/C (f) and corresponding EDX elemental spectrum (h), as well as the selected small bright spots (green square) from Pt + 5Au/C (g) and corresponding EDX elemental spectrum (i). Copper TEM grids were used. The histograms for the particle size distribution evaluation are based on an evaluation of ∼300 randomly chosen nanoparticles in the TEM micrographs of the respective as-prepared catalysts. The probability density of the particle size is plotted volume weighted and obtained from fitting of the SAXS data.

**Figure 2:**
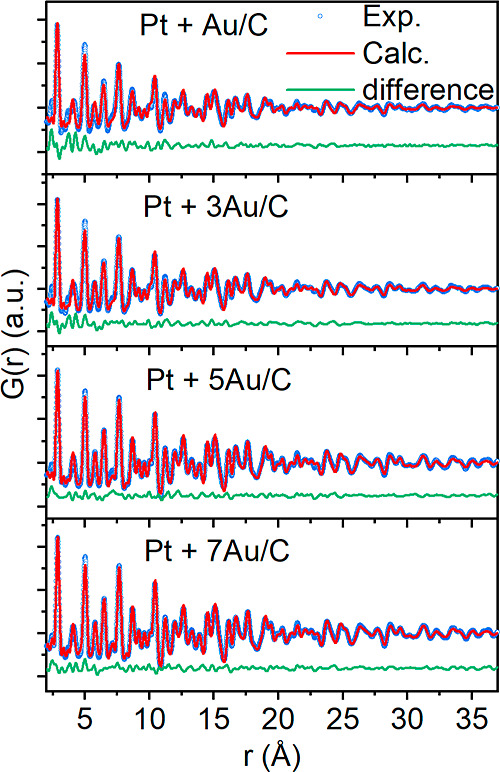
Fits to the PDF of the investigated pristine Pt + *x*Au/C nanocomposites. The fits are based on a two-phase model using both Pt and Au *fcc* structures. The blue circles show the experimental PDFs, the red lines are simulated PDFs, and the green lines are the difference curves of the two. Refined parameters are given in Table S3.

Pt–Au/C nanocomposites with varying ratios of Au and Pt nanoparticles distributed over the carbon support (the number of Pt nanoparticles, expressed as mass loading in wt %, is kept constant) were prepared from identical colloidal stock suspensions. The particle sizes of the Pt and Au colloids were chosen differently, that is, around 2 and 10 nm, respectively, to allow distinction. The obtained compositions were evaluated with ICP–MS and SEM–EDX (Table S1). In the following, the name Pt + *x*Au/C indicates the determined mass ratio of Pt to Au metal loadings. Transmission electron microscopy (TEM) and SAXS were used for evaluating the particle size distribution on the carbon support and one can observe that all the as-prepared samples exhibit two well-distinguishable size distributions (Figure 1a–d, the physical characterization of the Pt/C reference can be seen in Figure S1). Furthermore, the Pt and Au nanoparticles are clearly separated and uniformly distributed on the carbon support. Only at high Au contents, a slight aggregation of Au nanoparticles in the nanocomposites is indicated (Figure 1c,d, Table S2). In all nanocomposites, the smaller Pt nanoparticles are clearly discernible, and their particle size is not affected by the Au loading (Table S2). STEM EDX elemental mapping on Pt + 5Au/C allows in-depth characterization of the nanocomposites, that is, the large bright spots are related to the Au NPs (Figure 1f,h), while the smaller spots are related to Pt NPs (Figure 1g,i). No mixture of the elements is observed, which confirms that, in the pristine samples, the Pt and Au nanoparticles are immobilized separately on the carbon support. Due to the very small size (*ca.* 2 nm) of the Pt nanoparticles in the nanocomposites (Figure 1a–d), the XRD diffractograms only display clear Au Bragg peaks (Figure S2).^36^ The structural characterization of the nanocomposites is completed by total X-ray scattering.^37^ As illustrated in Figure 2, the pair distribution functions (PDFs) obtained from the four samples could all be described by a two-phase Au/Pt model, where smaller *fcc*-structured Pt nanoparticles (refined crystallite size *ca.* 2 nm) are identified along with larger *fcc*-structured Au nanoparticles (refined crystallite size *ca.* 5–6 nm). Note, however, that the similarities between the structure and scattering power of Pt and Au make it difficult to distinguish clearly between the two phases in the PDF, which leads to a large correlation between the refined parameter for the two phases. However, the existence of nanoparticles of two distinct crystallite sizes in the PDF is clear (Table S3), and the results are thus consistent with that from the SAXS and TEM analyses (Figure 1). In conclusion, Pt + *x*Au/C nanocomposites with variable compositions could be obtained with a monometallic nanoparticle preparation strategy.

### 2.2 Electro-oxidation of FA on the Investigated Catalysts under Potentiodynamic Conditions

**Figure 3:**
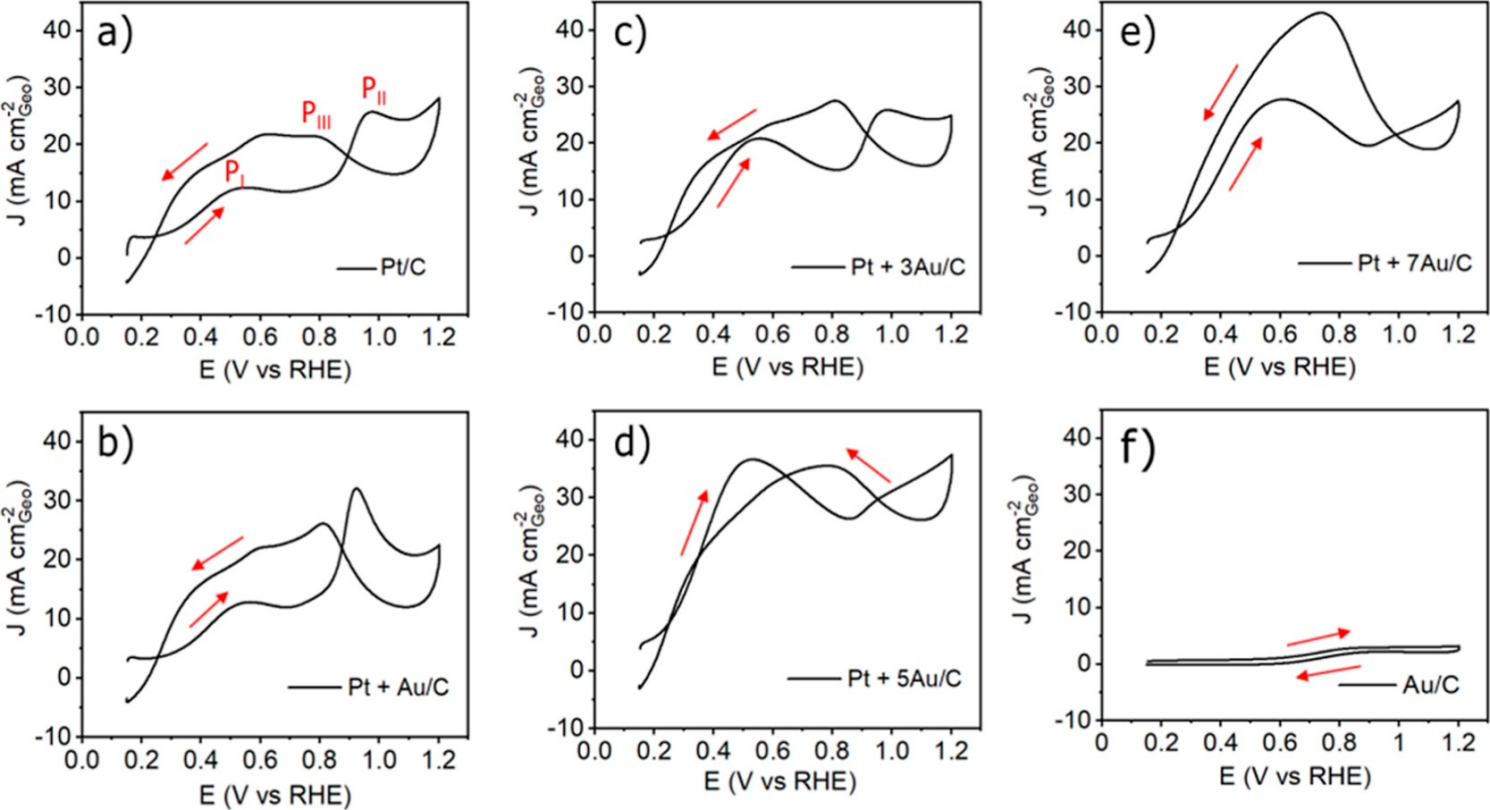
CVs of FAOR of the investigated catalysts recorded in the GDE setup. A gas bubbler connected to the GDE is filled up with 5.0 M formic acid and the upper cell body is filled up with 1.0 M HClO_4_. All CVs show the second scan from the potentiodynamic tests. A scan rate of 50 mV s^–1^ was applied for all measurements.

The electro-oxidation of FA on the investigated electrocatalysts is carried out in a GDE setup, an electrochemical testing platform using a Nafion membrane to mimic fuel cell operating conditions.^38−40^ FA is introduced to the GDE setup *via* FA-saturated Ar gas stream as described previously.^35^ The CVs from the potentiodynamic tests (Figure 3) provide fundamental information concerning the FAOR activity. According to the literature, several important characteristics can be used to describe the FAOR activity, that is, peak currents, peak potentials, and the hysteresis between positive- and negative-going scan; see Table 1 for a summary of the key performance characteristics of the different Pt + *x*Au/C nanocomposites (the values are extracted from Figure S3).^41^ High peak currents indicate high oxidation rates and low peak potentials or high currents at a low potential are a sign of a low overpotential. A small hysteresis indicates limited poisoning and a preferential direct oxidation pathway. A “good” catalyst, therefore, exhibits high peak currents, especially in the forward-going scan, low peak potentials, and a small hysteresis between the positive- and negative-going scan. The well-established features of a Pt reference catalyst for the FAOR can be seen in Figure 3a.^20,42^ The peak in the forward-going scan corresponding to the electro-oxidation of FA to CO_2_ through the direct pathway (dehydrogenation) exhibits only a weak intensity (P_I_). The peak with a strong intensity (P_II_) in the positive-going scan is associated with CO oxidation, which occurs *via* the indirect pathway (dehydration) and poisons Pt-based catalysts. Therefore, in catalyst design, one aims to increase the direct pathway (P_I_) and suppress the indirect path (P_II_) to avoid catalyst poisoning. By comparison, the current from pure Au/C is significantly smaller (Figure 3f) and pure Au seems to be inert to FAOR. However, in the case of the Pt + *x*Au/C nanocomposites, it is seen that the FAOR proceeds more through the direct pathway (Figure 3c–e, Pt + Au/C behaves rather similar to Pt/C, as indicated in Figure 3b). The peak currents at low potential (P_I_, as indicated on pure Pt/C) increase with the introduction of Au nanoparticles, while the peak currents at high potential (P_II_, as indicated on pure Pt/C) behave oppositely, that is, the P_II_ currents decrease (except for Pt + Au/C) and almost disappear on Pt + 5Au/C and Pt + 7Au/C. The accumulated FAO intermediates are oxidized at a high potential excursion; therefore, the peak currents indicated as P_III_ on pure Pt/C in the negative-going scan reflect the intrinsic activity of a “clean catalyst” towards the FAOR.^42^ One can see that as a result of the introduction of Au nanoparticles, the currents of P_III_ are enlarged as well, which implies that the electro-oxidation rates of FA are improved in comparison to monometallic Pt/C. In addition, a reduced hysteresis (based on P_I_ and P_III_ current) between both scan directions is observed on Pt + 3Au/C and Pt + 5Au/C (Figure 3c,d), reflecting the improved poisoning resistance. However, the hysteresis for Pt + Au/C (Figure 3b) and Pt + 7Au/C (Figure 3e) is rather similar to that of the Pt/C reference catalyst. All of these observations indicate that the performance of FAOR improves with the introduction of Au nanoparticles. The Au content of the nanocomposites thereby plays a significant role. Therefore, it is necessary to evaluate and compare the performance of FAOR quantitatively and systematically for the different Au contents.

**Table 1:** FAOR Characteristics of the Investigated Catalysts^a^

catalysts		Pt/C	Pt + Au/C	Pt + 3Au/C	Pt + 5Au/C	Pt + 7Au/C
forward scan	current at 0.3 V_RHE_ (A g_Pt_^–1^)	35.4 ± 5.4	48.8 ± 4.1	50.9 ± 6.2	98.4 ± 9.5	64.8 ± 5.9
	P_I_ current (A g_Pt_^–1^)	97.0 ± 4.1	152.0 ± 8.4	181.2 ± 6.2	286.6 ± 23.5	259.9 ± 25.5
	P_I_ potential (mV)	546.0 ± 1.7	562.3 ± 2.3	543.0 ± 4.6	516.0 ± 6.2	621.3 ± 17.2
backward scan	P_III_ current (A g_Pt_^–1^)	160.5 ± 25.9	322.4 ± 10.0	228.0 ± 13.3	287.2 ± 34.0	452.5 ± 28.1
	P_III_ potential (mV)	786.7 ± 5.9	831.0 ± 16.8	807.3 ± 2.9	780.3 ± 5.1	758.3 ± 21.5
hysteresis (from the current of P_I_ and P_III_, %)		39.0 ± 6.9	52.9 ± 1.4	20.1 ± 8.6	5.2 ± 2.4	42.4 ± 6.1
hysteresis (from the potential of P_I_ and P_III_, %)		30.6 ± 0.7	32.3 ± 1.2	32.7 ± 0.8	33.9 ± 1.1	18.0 ± 2.1

a The currents are evaluated and compared to each other at 0.3 V_RHE_ and at the peak positions P_I_ and P_III_ (as indicated in Figure 3a) from the positive-going and negative-going scans, respectively. Furthermore, the peak potentials (of P_I_ and P_III_) are compared and the hysteresis is calculated from the peak currents or peak potential (P_I_ and P_III_) of three different individual measurements determining the average value. The indicated errors are the standard deviation obtained from three independent measurements.

### 2.3 Performance Comparison of FA Electro-oxidation on the Investigated Catalysts

We scrutinize the FAOR performance based on the Pt mass-weighted currents, as Pt is the active phase for the FAOR (the actual metal mass on the GDL is displayed in Table S1). The values of the different characteristics are summarized in Table 1. A clear activity trend in peak current (P_I_) is seen from the positive-going scan. The electro-oxidation rate of FAOR on Pt/C is only 97.0 ± 4.1 A g_Pt_^–1^. With the introduction of Au nanoparticles, this value gradually increases to a maximum of 286.6 ± 23.5 A g_Pt_^–1^, a value that is ∼three times higher than the one from Pt/C, observed on Pt + 5Au/C. Further increasing the Au content (Pt + 7Au/C) does not lead to an additional improvement. However, instead, a maximum in activity from the backward scan (P_III_) is reached. A similar activity trend can also be observed at 0.3 V_RHE_, an anodic working potential in DFAFCs,^43^ in the forward-going scan. Apparently, the combination Pt + 5Au/C stands out, which is consistent with the lowest peak potential. In comparison to the reference catalyst, the value is ∼30 mV shifted to lower potentials. In addition, the current hysteresis of both directions, which reflects the poisoning resistance of a catalyst in FAOR, is compared. The hysteresis from pure Pt/C is ∼39%, whereas on Pt + 5Au/C it is reduced to only ∼5.2%, which indicates a substantially improved poisoning resistance and a preferential dehydrogenation path in the FAOR. By comparison, the nanocomposites with a much lower Au content (Pt + Au/C) or a higher Au content (Pt + 7Au/C) lead to a higher current in the forward scan but also to higher peak potentials and a higher or similar (Pt + 7Au/C) hysteresis. Taking into account the improved electrocatalytic performance for the FAOR with respect to the Au loading, the mass ratio of 1:5 (Pt/Au) displays a balanced performance improvement. Thus, we analyzed the Pt + 5Au/C catalyst further in *operando* SAXS, wide-angle X-ray scattering (WAXS), and identical location transmission electron microscopy (IL-TEM) studies.

### 2.4 Insights into the Improved FA Electro-oxidation Performance on Pt + *x*Au/C Nanocomposites

**Figure 4:**
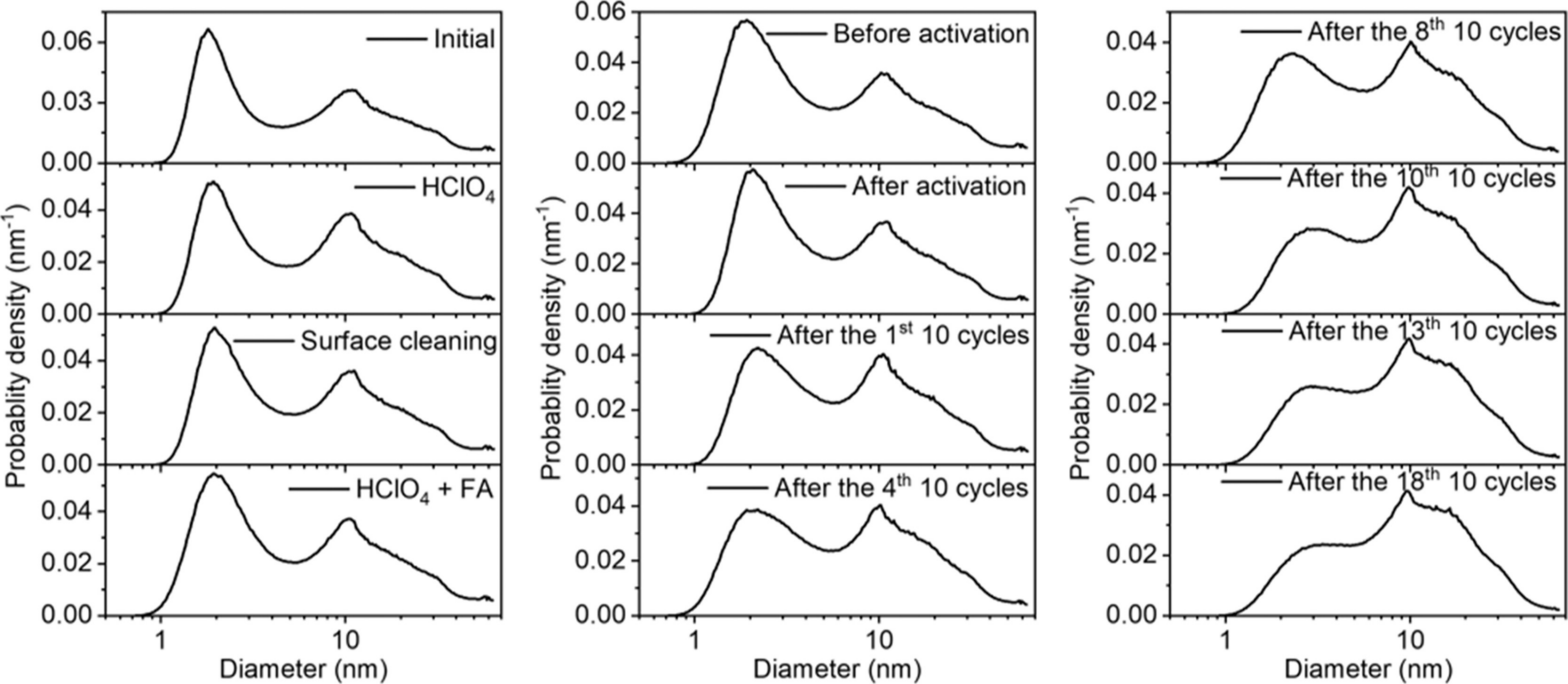
Size distributions of Pt + 5Au/C obtained from the analysis of *operando* SAXS data. The displayed size distribution functions are selected as a function of the duration of FAOR potentiodynamic tests. The first column shows the data before (indicated as initial) and after subjecting the catalyst to the electrolyte (HClO_4_), after applying a cleaning procedure, and after subjecting the catalyst to formic acid (FA). The next columns show the influence of the activation procedure and the potential cycling. In the FAO potentiodynamic test, the potential was swept between −0.042 and 1.008 V_Ag/AgCl_ with a scan rate of 50 mV s^–1^. The potential was held at 0.268 V_Ag/AgCl_ for 30 min to achieve catalyst activation. See experimental part for more details.

**Figure 5:**
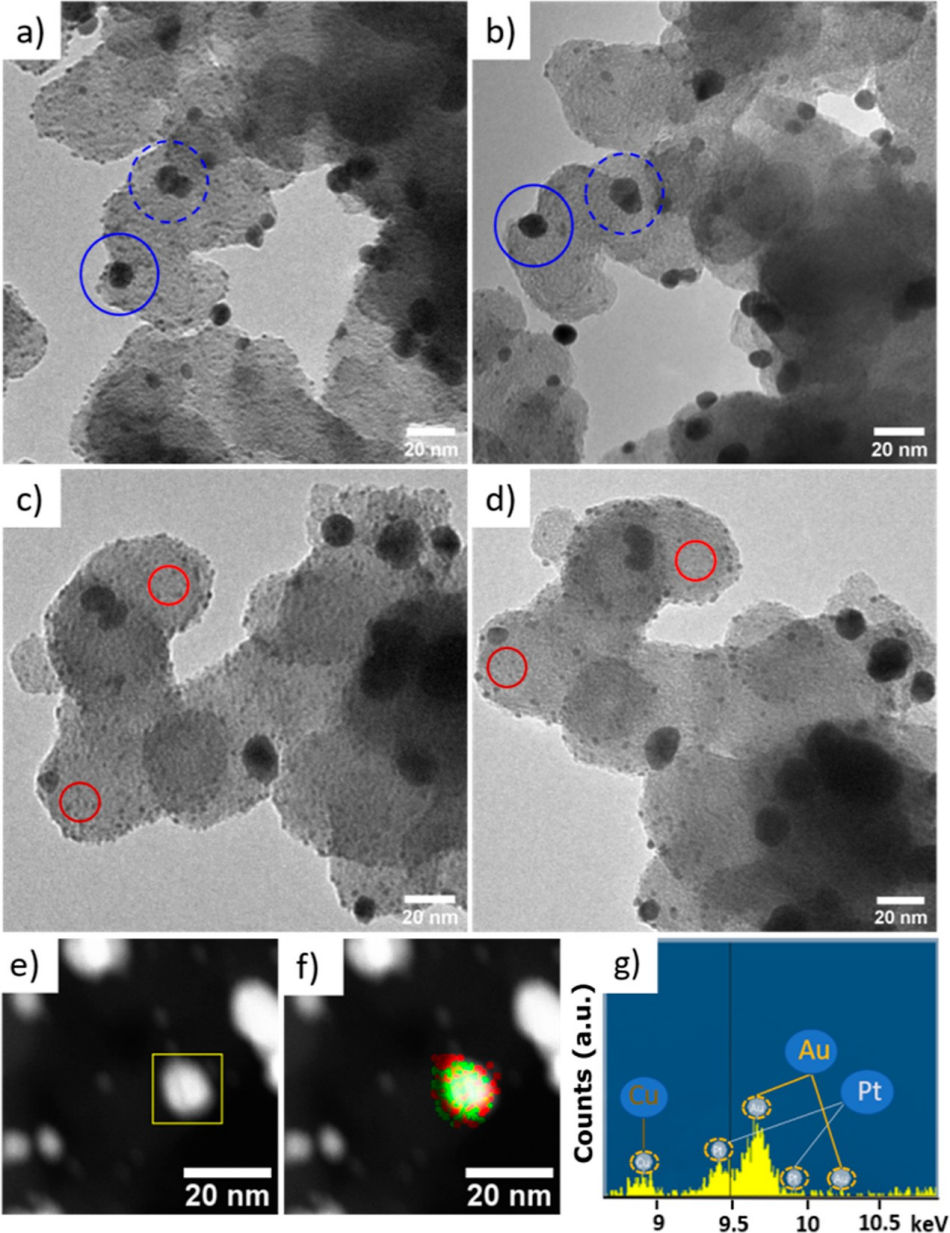
IL-TEM (a–d) and STEM micrographs (e), STEM–EDX mapping (f), and STEM–EDX elemental analysis (g) of the Pt + 5Au/C nanocomposite. The IL-TEM micrographs were obtained from the pristine Pt + 5Au/C sample (a,c), and the same sample exposed to 50 CVs in the FAOR potentiodynamic test (b,d). Red solid circles indicate shrinkage of Pt nanoparticles due to dissolution; blue solid circles indicate areas of suspected Pt nanoparticle migration and coalescence with Au islands, and blue dashed circles evidence Au nanoparticle migration and coalescence with Au nanoparticles (e). STEM–EDX elemental mapping (f) of Pt (red) and Au (green), and the corresponding EDX elemental spectrum (g) were collected after 50 CVs in the FAOR potentiodynamic test. The samples for STEM analysis were prepared by scraping catalyst powder from the GDL after the potentiodynamic test, followed by dispersing it into ethanol and dropping it onto a TEM copper grid.

**Figure 6:**
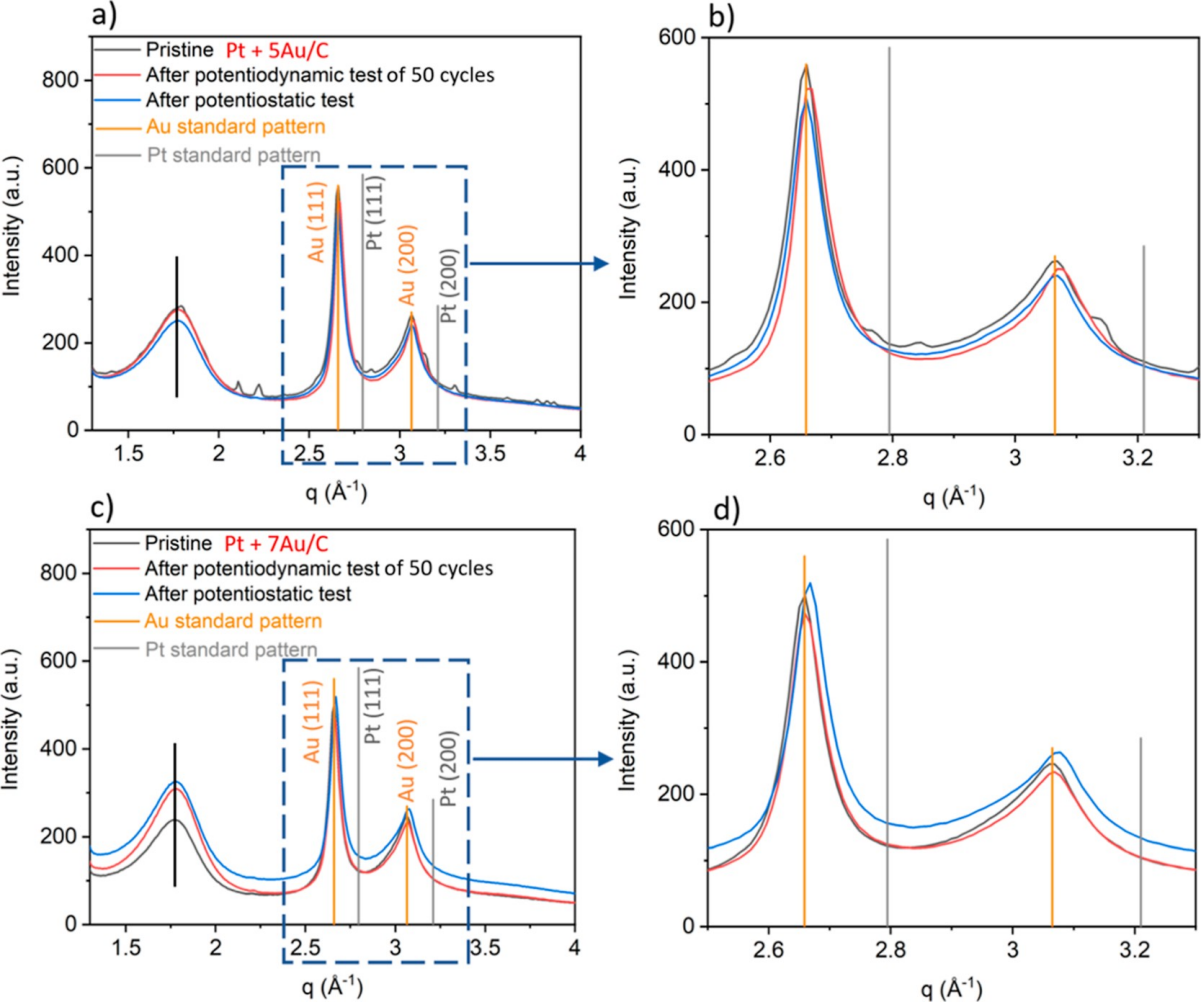
Profiles of WAXS diffractograms of Pt + 5Au/C (a,b) and Pt + 7Au/C (c,d). (b,d) Respective amplified diffraction patterns in the *q* range of 2.5–3.3 Å^–1^. 50 CVs were applied in the potentiodynamic test for Pt + 5Au/C and Pt + 7Au/C.

It has been documented that catalyst surfaces restructure under continuous potential cycling, for example, *via* metal dissolution and re-deposition, metal particle migration and coalescence, and so forth*.*^44,45^ Therefore, it was suspected that continuous potential cycling, as typically done in catalytic testing, could trigger the formation of Pt–Au alloys from the pristine nanocomposites. This hypothesis was tested based on the Pt + 5Au/C nanocomposite by combining *operando* SAXS, IL-TEM, STEM–EDX elemental mapping, and WAXS. The *operando* SAXS data were recorded during continuous FAOR measurements in potentiodynamic mode. While the analysis of the relative ratio between Pt and Au is challenging,^46^ the data clearly indicate a non-static behavior. It is seen that with cycling, the probability density associated with Pt NPs decreases in intensity and increases in size, indicating a gradual convergence of the two size populations associated with Pt and Au, respectively (Figures 4 and S4). The observed behavior would be in line with Pt dissolution and re-deposition onto Au islands as well as Pt NP migration and coalescence with Au nanoparticles during the continuous potential cycling. To further support our assumption, IL-TEM was used. A TEM Au grid deposited with Pt + 5Au/C was exposed to the same FAOR potentiodynamic conditions used in the GDE setup. In Figure 5a–d, the representative IL-TEM micrographs before and after potentiodynamic tests are displayed. As seen, IL-TEM indeed indicates signs of Pt nanoparticle migration and coalescence with Au nanoparticles as marked by the blue solid circles. Pt dissolution is evident by particle shrinking as demonstrated by the red solid circles, which is consistent with ICP–MS analysis—small amounts of Pt are detected in the electrolyte after potentiodynamic tests (Table S4), which might re-deposit onto the Au nanoparticles. Both processes lead to an *in situ* alloying of Pt and Au. This statement is further supported by STEM–EDX and WAXS. In STEM–EDX, alloy formation on the Au nanoparticles is difficult to distinguish from migration-coalescence (*i.e.*, alloying *vs* deposition of Pt on Au, Figure 5e–g); however, the WAXS data clearly indicate a small shift in the Bragg peak positions toward larger diffraction angles after the potentiodynamic test; see Figure 6a,b. Given that the Bragg peak positions from carbon (as indicated by the black vertical line) stay constant, that is, they can serve as an internal standard, the small shift in peak position indicates that Pt and Au form an alloy. According to the work of Guay *et al.*(32) studying Pt–Au alloy nanoparticles, the optimal (surface) composition of Pt–Au bimetallic catalysts for the FAO is ∼50% Pt. The optimal nanocomposite composition in this study is Pt + 5Au/C, that is, the mass contribution of Pt is roughly five times less than Au. This indicates surface enrichment of Pt, that is, a surface alloy of Pt and Au is formed on the Au NPs of the nanocomposite. Due to the similarity of Pt and Au, Rietveld refinement did not allow the unambiguous quantification of the formed alloy. However, on applying Vegard’s law,^47,48^ a fraction of ∼10% of alloying can be estimated. Taking into account that applying Vegard’s law only results in a rough estimation and does not account for surface alloying, one can assume an even higher degree of surface alloying. For comparison, for the nanocomposite with less Au content (Pt + 2Au/C, the corresponding CV in the potentiodynamic test displayed in Figure S5) was investigated with *operando* SAXS under the same conditions. The still well-distinguishable size populations (Figure S6) and minor changes in peak intensity ratio (Figure S7) at the end of the potentiodynamic tests indicate that *in situ* alloying of Pt and Au is substantially inhibited. This might be a simple consequence of a too low amount of Au, rendering the alloying less likely. This is further evidenced by analyzing the 50th CV of the potentiodynamic measurements. It is seen that the nanocomposites with low Au content still keep a similar feature of FAOR behavior, while a “more Pt–Au alloy” feature for the FAOR (an absence of P_II_ and a comparable P_I_ and P_III_ current from both directions) arises,^32^ especially on Pt + 5Au/C (Figure S8). Electronic and ensemble effects are normally predominant causes to lead to an improvement in FAO performance in the Pt–Au bimetallic system.^20,49,50^ However, by discussing the correlation between CO oxidation peak position and Au content (Figures S9 and S10) in the Supporting Information, one can conclude that the introduced Au does not allow faster removal of the CO intermediate. Therefore, the increased FAO activity of the surface alloy nanoparticles formed from Pt + 5Au/C is achieved by facilitating the direct path of FAO. This is most likely related to an ensemble effect, that is, Pt domains are interrupted by Au (neighboring Pt atoms are required for the formation of adsorbed CO)^20^ and thus suppressed CO adsorption on the Pt surface. The FA reaction environment plays an important part in the alloy formation. In Figure S11, IL-TEM micrographs of the Pt + 5Au/C nanocomposite exposed to potentiodynamic tests in different reaction environments are shown. Without organic molecules in the reaction environment, the effect of the electrochemical treatment on the catalyst is significantly reduced. In MeOH or EtOH environment, Pt detachment seems to be more predominant than in FA, leading to particle loss. However, the different organic reactants have not been investigated further.

### 2.5 Electro-oxidation of FA on the Investigated Catalysts under Potentiostatic Tests

**Figure 7:**
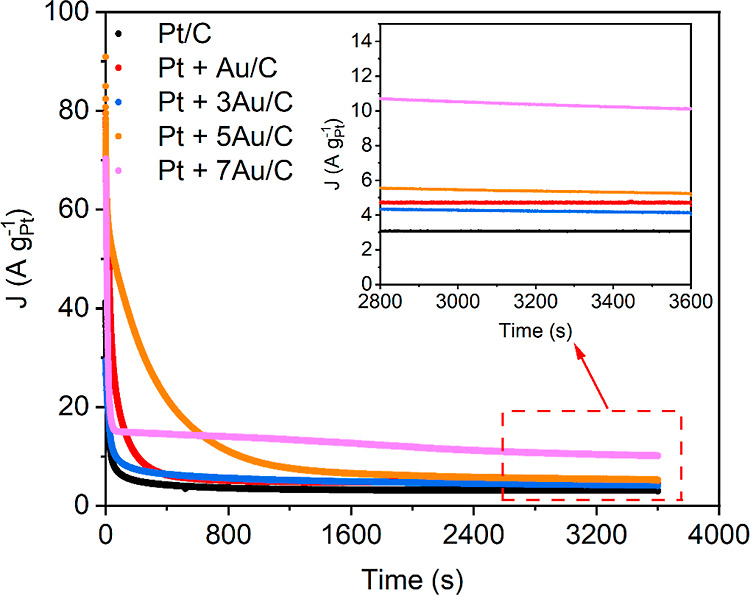
Potentiostatic measurements of FAOR holding the potential at 0.3 V_RHE_ for 1 h. The inset is the magnified view of catalytic activity at steady state.

Last but not least, the catalysts were also exposed to potentiostatic tests at 0.3 V_RHE_, which can be considered to simulate a steady-state DFAFC application. In these tests, a slightly different picture arises with respect to the relative performance of the catalysts (Figure 7). For all investigated catalysts, a substantial inhibition can be observed in the first 600 s. Pt + 5Au/C, which was the most active catalyst toward FAOR in the potentiodynamic tests, exhibits a much longer inhibition time than the reference Pt/C catalysts or the samples with lower Au content. However, in the long term, the sample suffers from the same performance inhibition. By comparison, the Pt + 7Au/C catalyst exhibits the best long-term performance stability. Analogous to the potentiodynamic measurements, it can be assumed that *in situ* alloying of Pt and Au nanoparticles takes place under FAOR potentiostatic conditions. This indicates that the mechanisms accounted for the *in situ* alloying are more pronounced in potentiodynamic treatments than in potentiostatic ones. Therefore, under potentiostatic conditions, a higher Au loading is required for the optimal result. This conclusion is supported by the particle size distributions obtained for Pt + 7Au/C from *ex situ* SAXS after the potentiostatic tests (Figure S12), which show signs of alloying between Pt and Au as well. Furthermore, also the Bragg peaks shift slightly in position (Figure 6c,d) as discussed before. By comparison, these changes are substantially less pronounced for the data of the Pt + 5Au/C catalyst, which display in the sample exposed to the potentiostatic tests similar peak positions as for the pristine sample (Figure 6a,b).

## 3 Conclusions

We present a concept of Pt + *x*Au/C nanocomposites with variable Au loadings for the FAOR. *Ex situ* and *operando* SAXS, WAXS, TEM, STEM–EDX, and PDF combined indicate that the separate deposition of Pt and Au nanoparticles onto a carbon support can be used to *in situ* form Pt–Au surface alloys with variable composition. Varying the Pt to Au ratio is easy and straightforward and requires only the preparation of two colloidal nanoparticle stock suspensions. This is a significant advantage over the elaborate preparation of series of alloy nanoparticles with varying composition. In our work, it is demonstrated how this nanocomposite approach can be used to optimize the FAOR performance. The optimization can be achieved not only *via* the Pt to Au ratio in the nanocomposite but also by varying the electrochemical conditioning. While in potentiodynamic tests Pt + 5Au/C is the optimal nanocomposite, steady-state conditioning indicates improved long-term activity for Pt + 7Au/C. Although the exact amount of surface alloying is difficult to differentiate for the Pt–Au system, the results indicate that as compared to alloy nanoparticles, the nanocomposite concept does not only allow for an easier preparation and optimization, it might also show a way to optimize the use of precious and critical raw materials by surface alloying, in our case Pt onto Au nanoparticles.

## 4 Experimental Section

### 4.1 Chemicals and Materials

The following chemicals were used for platinum nanoparticles (Pt NPs) synthesis and flocculation: hexa-chloroplatinic (IV) acid hexahydrate (H_2_PtCl_6_·6H_2_O, 99.9%, Alfa Aesar), ethylene glycol (EG, 99%, Alfa Aesar), sodium hydroxide (NaOH, 99.99%, Merck), 37% hydrochloric acid (HCl, Suprapur, Merck), and acetone (for HPLC, VWR Chemicals BDH). Vulcan XC72R carbon black was employed as support for the metal nanoparticle deposition. 37% hydrochloric acid (HCl, Suprapur, Merck) and 65% nitric acid (HNO_3_, Suprapur, Merck) were used for the dissolution of the metal nanoparticles for inductively coupled plasma mass spectrometry analysis. Isopropanol (IPA, for HPLC, VWR Chemicals BDH), Milli-Q water (resistivity > 18.2 MΩ·cm, total organic carbon (TOC) < 5 ppb), and Nafion (D1021, 10 wt %, Fuel Cell Store) were used for catalyst ink preparation. 70% perchloric acid (HClO_4_, Suprapur, Merck) and formic acid (FA, ≥95%, Sigma-Aldrich) were used for the preparation of the electrolyte and reactant during the electrochemical measurements. Gas diffusion layers (GDL) with microporous layer (H23C8, Freudenberg) and without microporous layer (H23, Freudenberg) as well as Nafion membranes (Nafion 117, Fuel Cell Store) were used for the catalyst layer assemblies. Ar (99.999%, Air Liquide) and CO (99.97%, Air Liquide) were used for the electrochemical measurements.

### 4.2 Synthesis of Supported Pt/C and Pt + *x*Au/C Nanocomposite Catalysts

The synthesis of the nanocomposite catalysts consisted of two steps: the preparation of colloidal metal NPs and the supporting onto the carbon black. A laser-based approach was applied to prepare colloidal Au NPs (suspension in water). The specific method was detailed in previous studies.^51,52^ For the preparation of the Pt NPs, a surfactant-free colloidal approach was applied,^53^ which was outlined in our recent work.^33,34^ Briefly, the same volumes of NaOH EG solution (400 mM) and H_2_PtCl_6_·6H_2_O EG solution (40 mM) were mixed and heated to 160 °C in a microwave reactor for 3 min to obtain colloidal Pt NPs in EG with a concentration of 3.90 g_Pt_ L^–1^. To support the Pt NPs onto carbon, the Pt colloid in EG was flocculated with 1 M HCl and centrifuged, whereafter the Pt NPs could be separated from the EG and re-dispersed in acetone (3.90 g_Pt_ L^–1^). Then, the carbon support (Vulcan XC72R) acetone suspension was mixed with a specific amount of Pt NPs acetone suspension and homogenized by a horn sonicator for 5 min. The acetone was evaporated with a rotary evaporator obtaining 10 wt % Pt/C (nominal loading) catalyst powder. To prepare supported Pt + *x*Au/C nanocomposite catalysts, the Pt NPs acetone suspension (the Pt content was the same as for Pt/C) and Au NPs water suspension (0.075 g_Au_ L^–1^) were added to the carbon acetone suspension as simultaneously as possible under vigorous stirring. The mixture was left in the fume hood overnight under mild stirring for solvent evaporation to obtain a dried catalyst powder. The same preparation approach was applied for all Pt + *x*Au/C nanocomposite catalysts keeping the nominal Pt content constant while varying the Au content.

### 4.3 Electrochemical Measurements in the GDE Setup

All electrochemical measurements were performed in a GDE setup with a three-electrode configuration at room temperature. A computer-controlled potentiostat (ECi-200, Nordic Electrochemistry ApS) was employed. The in-house developed GDE setup is detailed in the previous studies.^38−40^ Briefly, the GDE (working electrode) was placed on the top of the lower cell body, which is made of polytetrafluoroethylene (PTFE), onto which the upper cell body made out of PTFE was assembled. A Pt mesh was used as a counter electrode (CE), and a reversible hydrogen electrode (RHE) was used as a reference electrode (RE). They were placed in the upper cell body filled with 1 M HClO_4_ as the electrolyte. A bubbler filled up with 5 M FA was used to bring the reactant to the catalyst using Ar gas for purging. The FA-enriched gas stream was introduced to the GDE as described previously.^35^

For the GDE fabrication, the catalyst (200 μg_Pt_ cm_geo_^–2^ is the nominal loading for all catalysts) was deposited onto the GDL (H23C8) *via* vacuum filtration.^54^ Ø 3 mm of the filtered catalyst layer was punched and inserted to a Ø 2 cm GDL (H23C8) with a hole in the center, and the Nafion membrane (Nafion 117) of 1.5 cm in diameter was pressed on top of the GDL.

Prior to the electrochemical measurements, the catalyst surface was cleaned by cycling in a potential region of 0.15–1.20 V_RHE_ with the scan rate of 500 mV s^–1^, until a stable cyclic voltammogram (CV) could be detected (normally 20–30 cycles). During the cleaning procedure, the gas bubbler was filled up with Milli-Q water. Thereafter, CO stripping measurements were conducted on each investigated catalyst to determine the accessible Pt surface area (ECSA). Thereafter, the Milli-Q water in the gas bubbler was replaced by 5 M FA and Ar was flushed for 10 min (we tested different concentrations of FA in the gas bubbler, see Figure S13), followed by a potential hold at 0.46 V_RHE_ for 30 min for catalyst activation. Potentiodynamic tests (potential cycling in the region of 0.15–1.20 V_RHE_ for ∼50 cycles with a scan rate of 50 mV s^–1^) were performed to obtain the most representative CV (normally a stable CV could be obtained within the first 20 cycles).

A similar measurement procedure was performed in the potentiostatic tests, that is, catalyst surface cleaning, CO stripping measurement, and catalyst activation. The potentiostatic tests were then conducted at a static potential of 0.30 V_RHE_ for 1 h. The corresponding current was recorded as a function of time to evaluate the poisoning resistance of the investigated catalysts.

### 4.4 Characterization

#### 4.4.1 *Ex Situ* SAXS and WAXS

The *ex situ* measurements were performed at the Niels Bohr Institute of the University of Copenhagen using a Nano-inXider instrument from Xenocs (Grenoble, France) using a Cu Kα source with a 1.54 Å wavelength and a two-detector setup for simultaneous SAXS/WAXS measurements. Both detectors are Pilatus3 hybrid pixel detectors from Dectris. Using a 0.8 mm beam size, the system provides a combined *q*-range from 0.01 to 4 Å^–1^ with a flux on the sample of *ca.* 60 Mph/s. Samples were measured for 30 min. The samples with metal NPs on the GDL were placed between two mica windows in dedicated sandwich cells, and the background was measured on a same GDL plus carbon black (without metal NPs). All the data fitting was conducted with a home-written MATLAB code assuming log-normal size distributions. The size distribution is shown as total volume weighted probability density. The specific expressions used in the MATLAB code are detailed in previous reports.^34^ All the values obtained for the free parameters in the model and the corresponding fits are shown in Table S2 and Figure S14, respectively.

#### 4.4.2 *Operando* SAXS

The measurements were carried out at the cSAXS beamline at SLS, PSI (Villigen, Switzerland). The *operando* cell was adapted from Binninger *et al.*(55) and is detailed in our previous studies.^56^ In contrast to the GDE setup, the electrode is directly in contact with the liquid electrolyte. A GDL (H23C8; Freudenberg) served as the CE, Ag/AgCl served as the RE, and a pre-filtered catalyst layer (punched a circle piece of 5 mm in diameter) inserted in a GDL stripe (a hole of 5 mm in diameter in the middle) served as the WE. Both CE and WE were fixed on a Kapton tape with the MPL side facing the electrolyte (0.1 M HClO_4_ or 0.1 M HClO_4_ + 0.1 M FA). The electrolyte was pumped into the cell with a syringe pump (KD Scientific) throughout the measurement, and the flow rate of electrolyte was kept at 1 mL min^–1^. The electrochemistry measurements were conducted in the following sequence: HClO_4_ introduction, potential sweeping for catalyst surface cleaning (−0.042–1.008 V_Ag/AgCl_ with a scan speed of 500 mV s^–1^, 25 cycles), HClO_4_ + FA introduction, potential cycling (−0.042–1.008 V_Ag/AgCl_ with a scan speed of 50 mV s^–1^, 10 repeats), catalyst activation (holding potential at 0.268 V_Ag/AgCl_ for 30 min), and potentiodynamic test with potential cycling (−0.042–1.008 V_Ag/AgCl_ with a scan rate of 50 mV s^–1^, 10 cycles is a set, 18 and 10 sets in total for Pt + 5Au/C and Pt + 2Au/C, respectively). The pristine sample prior to any electrochemical measurements was measured, and then, after each electrochemistry step, *operando* SAXS was conducted to monitor the change in particle size distribution. For the potentiodynamic FAOR test, after each set of potential cycling, *operando* SAXS measurements were collected. As schemed in Figure S15, four different spots on the GDL were monitored for background correction and two spots on the catalyst layer for sample analysis. Sample and background spots were measured in sequence by shifting between the *x*–*y* stage of each collected scattering data in each run. The “background” experienced the same electrochemical treatment as the sample in each run to avoid artifacts.^57^ All electrochemistry measurements were performed with unpurged electrolyte solutions.

A home-written program was used for *operando* SAXS analysis. All SAXS fittings were conducted using a Monte Carlo approach without introducing subjective factors. Before fitting, the data from two different spots for catalyst measurements and the data from the corresponding spots for the background measurement were averaged in the program. The following parameters were applied for *operando* SAXS analysis: error weighing of q (for Pt + 5Au/C) and q^2^ (for Pt + 2Au/C), smoothing of no weight, norm after iteration of 1000, and number of smoothings of 50; the particle sizes were logarithmically scaled.

#### 4.4.3 TEM and IL-TEM

The TEM analysis was conducted at the Microscopy Imaging Center (MIC) at the University of Bern. A FEI Tecnai Spirit transmission electron microscope operated at 80 kV and equipped with an Olympus-SIS Veleta CCD Camera was used for recording the micrographs. The as-prepared catalyst samples were prepared by dispersing catalyst powder in ethanol and dropping the catalyst suspension onto TEM copper grids. The samples exposed to electrochemical measurements and potentiostatic tests were collected by scraping the catalysts off the GDLs and dispersing them in ethanol. TEM micrographs were collected from five independently selected areas for each analyzed sample.

The same equipment and a gold finder grid were used for the IL-TEM analysis. Concerning sample preparation, the Pt + 5Au/C catalyst powder was dissolved in the mixture of IPA and H_2_O (*V*_IPA_/*V*_H_2_O_ = 3:1), and 7 μL of Nafion was added to lead to the weight ratio between Nafion and carbon support of 1:1. The catalyst ink was diluted by a factor of 10, and 10 μL of the diluted ink was pipetted onto a Au grid for IL-TEM analysis before electrochemistry. Afterward, the same Au grid was placed between a Nafion membrane and GDL in the GDE setup, and after the FAOR potentiodynamic test, the Au grid was analyzed with IL-TEM.

#### 4.4.4 Scanning Electron Microscope (SEM)

The composition of the filtered Pt + *x*Au/C nanocomposites on GDL was determined by SEM–EDX. The Ø 3 mm GDL stuck on the carbon tape was placed on a metal holder for SEM analysis. The analysis was performed on a Zeiss Gemini 450 SEM, which is equipped with an Oxford Instruments UltimMax 65 EDX detector, and a voltage of 25 kV was used for all SEM measurements. Five random areas on the GDL were determined for each catalyst. A software of AZtec 4.2 was used to acquire the EDX spectra and to analyze the catalyst composition.

#### 4.4.5 Scanning Transmission Electron Microscopy (STEM)

STEM imaging in combination with EDX analysis was carried out by using a 3000F JEOL transmission electron microscope equipped with a scan unit for STEM and an Oxford instruments SiLi-detector. An analytical probe with a nominal size of 1 nm and a 70 μm condenser aperture were used to maximize the EDS signal. A high-angle annular dark-field (HAADF) detector was used for imaging. The as-prepared catalyst samples were prepared by dispersing catalyst powder in ethanol and dropping the catalyst suspension onto TEM copper grids. The samples exposed to FAO potentiodynamic measurements tests were collected by scraping the catalysts off the GDLs and dispersing them in ethanol.

#### 4.4.6 Pair Distribution Function (PDF)

X-ray total scattering data for pair distribution function analysis were collected at the 11-ID-B beamline at the Advanced Photon Source, Argonne National Laboratory, Argonne, USA. All data were collected at room temperature using a wavelength of 0.2113 Å. The samples were measured in between two layers of Kapton films.

The detector distance and geometrical parameters were obtained from calibration in Fit2D,^58^ and the 2D patterns were integrated using pyFAI in Dioptas.^59,60^ The scattering signal from the carbon background was subtracted from the total scattering data before obtaining PDFs. The total scattering data were Fourier transformed to obtain the PDF using PDFgetX3^61^ and modeled using PDFgui.^62^ The following parameters were used in PDFgetX3: Qmin = 0.1 Å^–1^, Qmax = 18 Å^–1^, Qmax_inst_ = 25 Å^–1^, and rpoly = 0.9 Å.

The PDF was modeled with a two-phase model using the Pt *fcc* and Au *fcc* structures. The refinements were done in the *r*-range of 1.5–60 Å. In the refinement of the model to the PDF from the Pt + Au/C sample, the scale factor, unit cell parameter, and crystallite size for each of the two phases were refined. An isotropic atomic displacement parameter and a parameter describing correlated motion (delta2) were furthermore refined, however, these were constrained to take the same value for the two phases due to high correlation. For the rest of the Pt + *x*Au/C nanocomposite samples, where Pt is the minority phase, the refinement was done in a similar way, except that the unit cell parameter for Pt was kept fixed at the value obtained for the Pt + Au/C sample. Crystallite sizes of each sample were obtained from real-space Rietveld refinement by taking into account instrumental broadening. The refined parameters can be found in Table S3.

#### 4.4.7 Inductively Coupled Plasma Mass Spectrometry (ICP–MS)

The actual metal contents after vacuum filtration on the GDL were determined by ICP–MS (NexION 2000 ICP–MS), which is equipped with a cyclonic spray chamber and a PFA-nebulizer. The samples were prepared by immersing the Ø 3 mm circle GDL with filtered catalyst into aqua regia (volume ratio of HCl/HNO_3_ = 3:1) overnight, and the solution was then diluted to 200 mL with Milli-Q water for ICP–MS analysis.

## Supporting Information Available

Physical characterization of Pt/C reference; composition determination of nanocomposites; SAXS data fits and size analysis of the pristine catalysts; XRD patterns of pristine catalysts; PDF refinement; CO stripping measurements; quantitative analysis extracted from *operando* SAXS measurements; CVs of FAOR potentiodynamic tests after a certain repeat and with different FA concentrations; and sketch of WE for *operando* SAXS measurements (PDF)
