# Supplementary material for: Nanocomposite Concept for Electrochemical In Situ Preparation of Pt–Au Alloy Nanoparticles for Formic Acid Oxidation
Source: JACS Au. 2022 Jul 6;2(7):1757–68. doi: 10.1021/jacsau.2c00335 (PMC9327087; doi:10.1021/jacsau.2c00335)
Supplement: Supplementary file 1 — au2c00335_si_001.pdf [file au2c00335_si_001.pdf]

Supporting Information

Nanocomposite Concept for Electrochemical *in situ* Preparation of Pt-Au Alloy Nanoparticles for Formic Acid Oxidation

Jia Du[a], Jonathan Quinson[b,c], Damin Zhang[a], Baiyu Wang[b], Gustav K.H. Wiberg[a], Rebecca K. Pittkowski[b], Johanna Schröder[a], Søren B. Simonsen[d], Jacob J. K. Kirkensgaard[e,f], Yao Li[g], Sven Reichenberger[g], Stephan Barcikowski[g], Kirsten M. Ø. Jensen[b], Matthias Arenz[a] *

[a] Department of Chemistry, Biochemistry and Pharmaceutical Sciences, University of Bern, 3012 Bern, Switzerland

[b] Department of Chemistry, University of Copenhagen, 2100 Copenhagen, Denmark

[c] Department of Biochemical and Chemical Engineering, University of Aarhus, 8200 Aarhus, Denmark

[d] Department of Energy Conversion and Storage, Technical University of Denmark, 2800 Lyngby, Denmark

[e] Department of Food Science, University of Copenhagen, 1958 Frederiksberg, Denmark

[f] Niels-Bohr-Institute, University of Copenhagen, 2100 Copenhagen, Denmark

[g] Technical Chemistry I and Center of Nanointegration Duisburg Essen (CENIDE), University of Duisburg-Essen, 45141 Essen, Germany

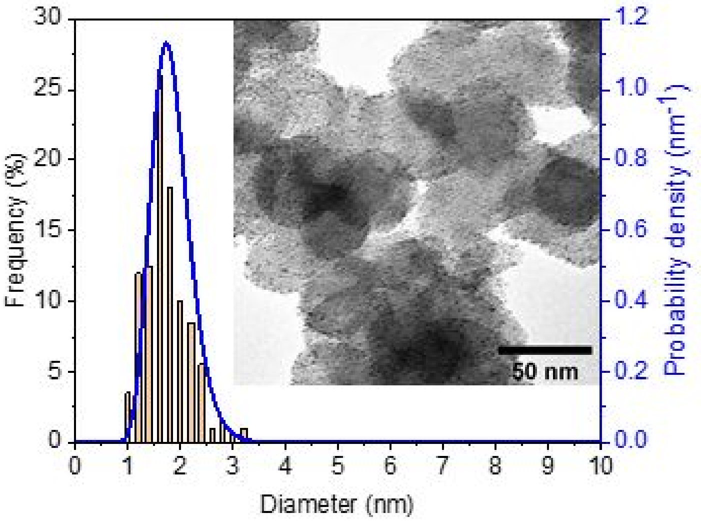

Figure S1. TEM micrographs and size distribution of the Pt/C reference. The histograms for particle size distribution evaluation are based on a statistical analysis of 200 Pt particles of the as-prepared Pt/C catalysts from TEM micrograph and the volume weighted probability density of the particle size is derived from SAXS measurement.

Table S1. The actual metal loading on GDL after vacuum filtration and the respective mass ratio between Au and Pt of the investigated electrocatalysts. The actual Pt and Au loading on GDL are determined by ICP-MS, the mass ratio of Au:Pt is checked by SEM-EDX and calculated from ICP-MS results, respectively.

	Pt mass on GDL (µg)	Au mass on GDL (µg)	Au:Pt (ICP-MS)	Au:Pt (SEM-EDX)
Pt/C	8.8	-	-	-
Pt + Au/C	5.6	5.8	1.04	1.10
Pt + 3Au/C	8.3	21.9	2.64	3.03
Pt + 5Au/C	8.3	39.3	4.73	5.33
Pt + 7Au/C	7.1	52.1	7.34	6.82
Au/C	-	6.0	-	-

Table S2. Parameters of SAXS data fits and size analysis of the pristine samples.

Catalysts	Power law		1st population			2nd population			Size and distribution							
									Smaller NPs 'Pt'			Larger NPs 'Au'			Overall	
	A x 10^6	n	R_1 (Å)	σ_1	C_1	R_2 (Å)	σ_2	C_2	D_1 (nm)	$\sigma_{1'}$ (nm)	Volume fraction 1	D_2 (nm)	σ_2 (nm)	Volume fraction 2	Overall Diameter D (nm)	Overall Standard Deviation of D σ (nm)
Pt/C	25	3.5	9.0	0.20	0.0055	27.0	0.30	0	1.8	0.4	1.00	-	-	0	1.8	0.4
Pt + Au/C	23	3.5	9.0	0.20	0.0020	40.0	0.25	0.01	1.8	0.4	0.81	8.3	2.1	0.19	3.0	0.4
Pt + 3Au/C	50	3.8	9.5	0.15	0.0022	44.0	0.25	0.05	1.9	0.3	0.53	9.1	2.3	0.47	5.3	1.1
Pt + 5Au/C	35	4.0	9.5	0.15	7E-4	49.0	0.25	0.05	1.9	0.3	0.31	10.1	2.6	0.69	7.6	1.8
Pt + 7Au/C	50	4.0	8.5	0.10	7E-4	48.0	0.25	0.05	1.7	0.2	0.36	9.9	2.5	0.64	6.9	1.6

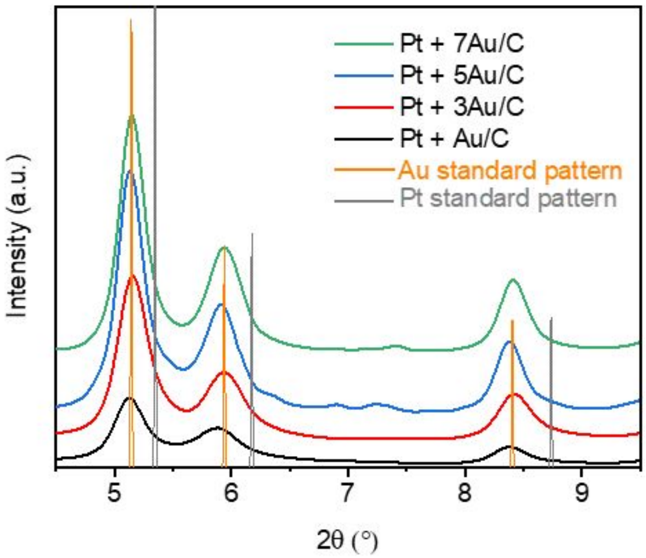

Figure S2. XRD diffractograms of the pristine Pt + xAu/C nanocomposites. The results are based on the total scattering data the PDFs were obtained from.

Table S3. Results from PDF refinements.

Catalysts	Au unit cell (Å)	Pt unit cell (Å)	Au/Pt Uiso (Å^2)	Au/Pt delta2 (Å)	Au crystallite size (Å)	Pt crystallite size (Å)	Au mass fraction	Pt mass fraction	Rw (%)
Pt + Au/C	4.09	3.97	0.010	4.3	50	17	0.65	0.35	26.4
Pt + 3Au/C	4.10	3.97	0.010	4.5	50	20	0.75	0.25	18.2
Pt + 5 Au/C	4.10	3.97	0.009	4.3	62	21	0.85	0.15	13.3
Pt + 7Au/C	4.11	3.97	0.010	4.8	63	17	0.72	0.28	12.5

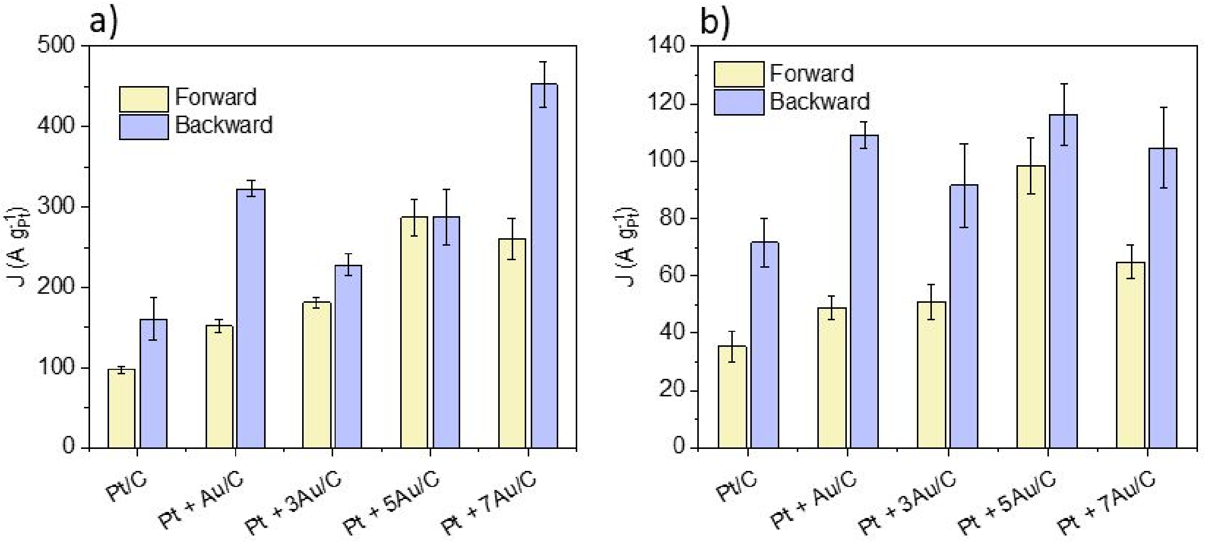

Figure S3. FAOR performance comparison of the investigated electrocatalysts with oxidation current recorded at (a) peak position (P_I for forward scanning and P_{III} for backward scanning, as displayed in Figure 3 in main text) and (b) potential of 0.3 V_{RHE} from both scanning directions. The standard deviation is obtained from at least three independent measurements for each investigated catalyst.

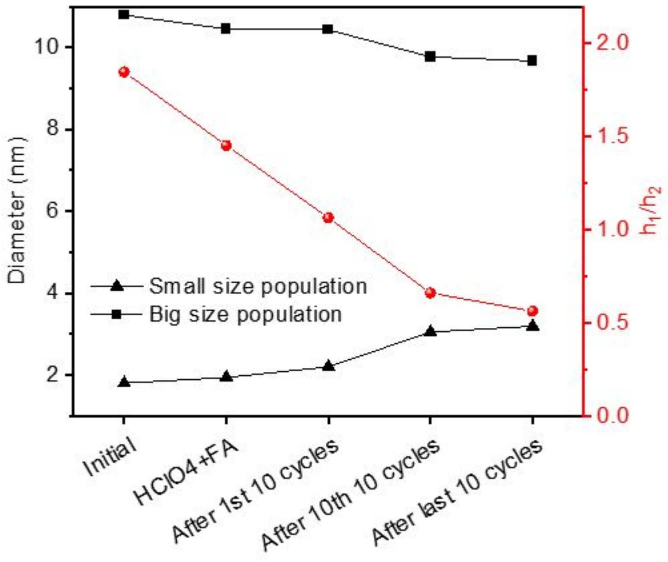

Figure S4. Particle characteristics of Pt + 5Au/C extracted from *operando* SAXS analysis. The corresponding particle size distributions are displayed in Figure 4 in the main text. The particle diameters of both populations reported here are based on the respective peak positions of maximum probability (mode) retrieved from the distribution functions presented in Figure 4. The h_1/h_2 ratios are calculated from the relative peak intensities at the mode for the small and big size populations on distribution functions.

Table S4. Pt and Au mass in 1.0 M $HClO_4$ electrolyte determined by ICP-MS. The electrolyte is filled in upper cell body of GDE setup. 5 mL of electrolyte in upper cell body is used for ICP-MS test before and after potentiodynamic test, respectively.

Pt + 5Au/C	Pt mass in electrolyte (μg)	Au mass in electrolyte (μg)
Before potentiodynamic test	0.0080	0.0000
After potentiodynamic test	0.1470	0.0009

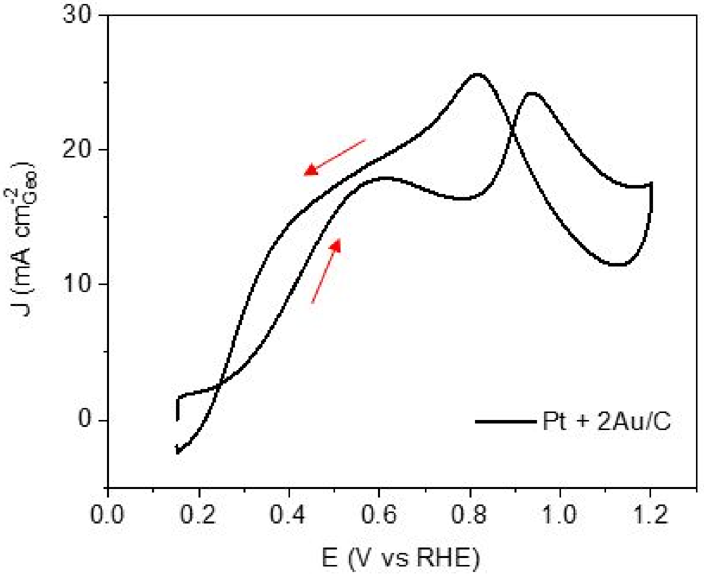

Figure S5. CV of FAOR potentiodynamic test of Pt + 2Au/C nanocomposite in GDE setup. The bubbler is filled up with 5.0 M formic acid and the upper cell body is filled up with 1.0 M $HClO_4$. A scanning speed of 50 mV s^{-1} is applied for measurements.

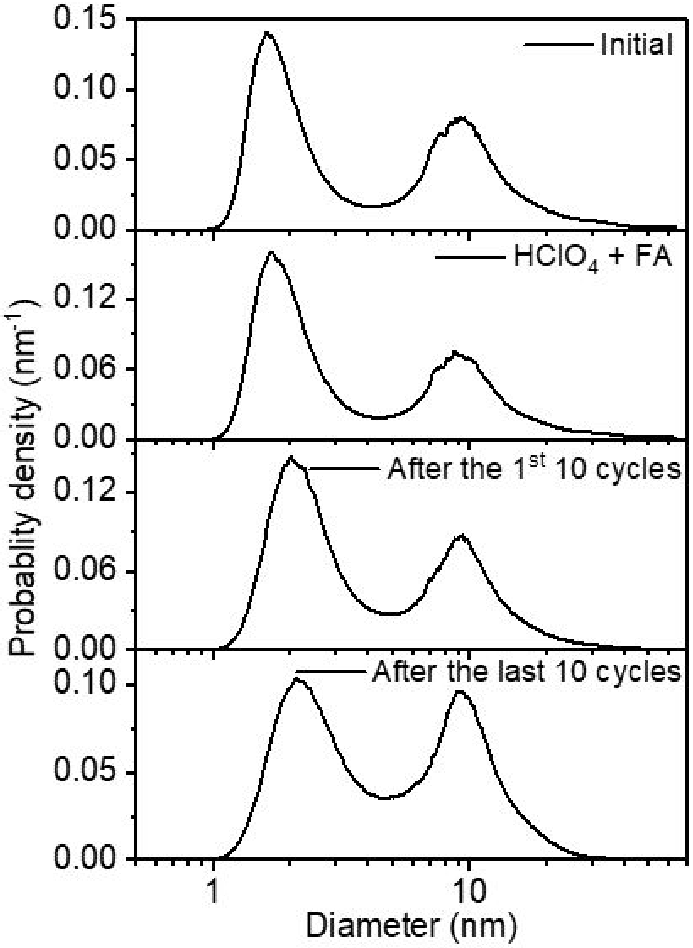

Figure S6. Size distributions of Pt + 2Au/C from operando SAXS analysis. The displayed size distribution functions are selected after a certain step of FAOR potentiodynamic test and in an order of top to the bottom as proceeding sequential measurement steps. A pure GDL serves as background for each background subtraction. Background and sample data are respectively averaged before SAXS fitting.

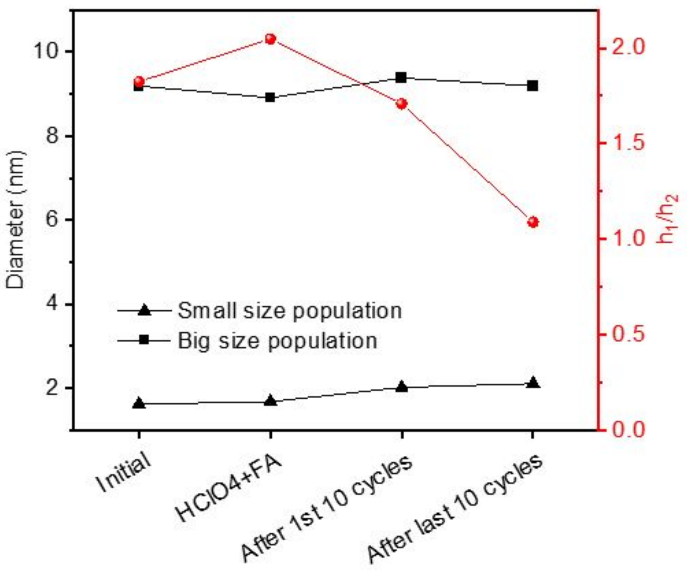

Figure S7. Particle characteristics of Pt + 2Au/C extracted from *operando* SAXS analysis. The corresponding particle size distributions are displayed in Figure S6. The particle diameters of both populations reported here are based on the respective peak positions of maximum probability (mode) retrieved from the distribution functions presented in Figure S6. The h_1/h_2 ratios are calculated from the relative peak intensities at the mode for the small and big size populations on distribution functions.

It is necessary to point out that less cycles were done in potentiodynamic test on Pt + 2Au/C. Ten times of potential cycling in each repeat and ten repeats in total. The last 10 cycles is corresponded to the 10th 10 cycles on Pt + 2Au/C. It is seen that the ratio value of h_1/h_2 is decreased from 1.82 the initial value to 1.09 after potentiodynamic test, by comparison, h_1/h_2 value is decreased from 1.85 to 0.66 after the 10th 10 potential sweeping on Pt + 5Au/C.

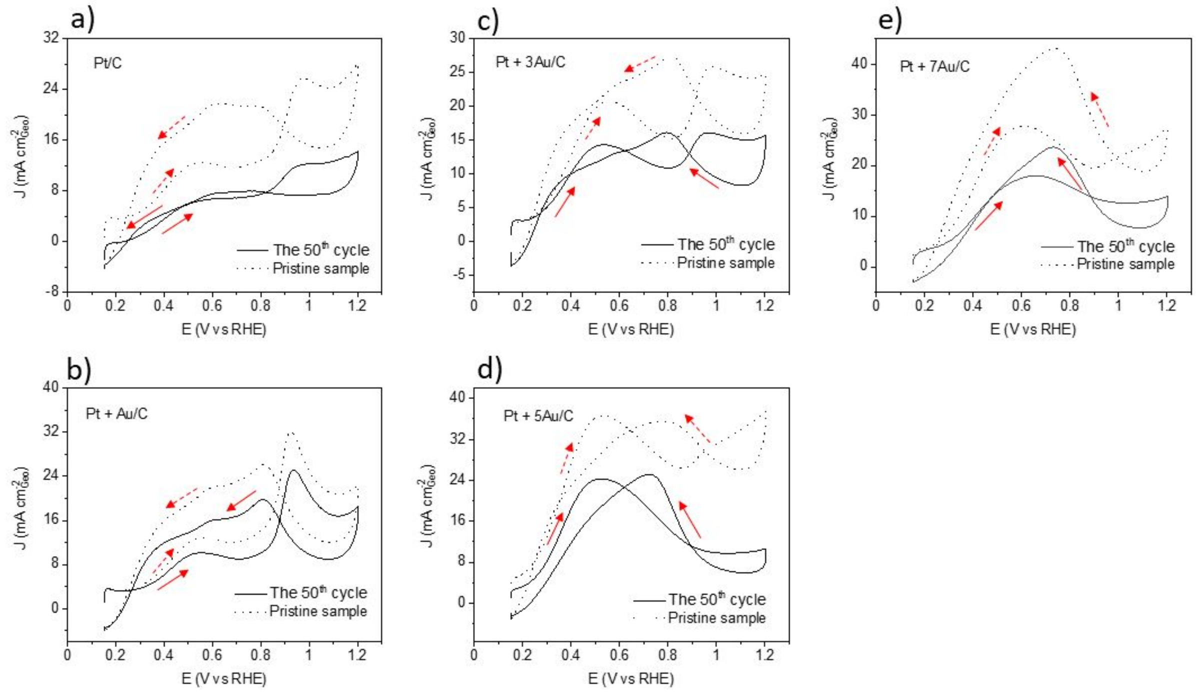

Figure S8. Comparison of CVs recorded from the pristine sample and the samples experience 50 times of potential sweeping in the FAOR potentiodynamic test in GDE setup. The bubbler is filled up with 5.0 M formic acid and the upper cell body is filled up with 1.0 M $HClO_4$. A scanning speed of 50 mV s^{-1} is applied for all measurements.

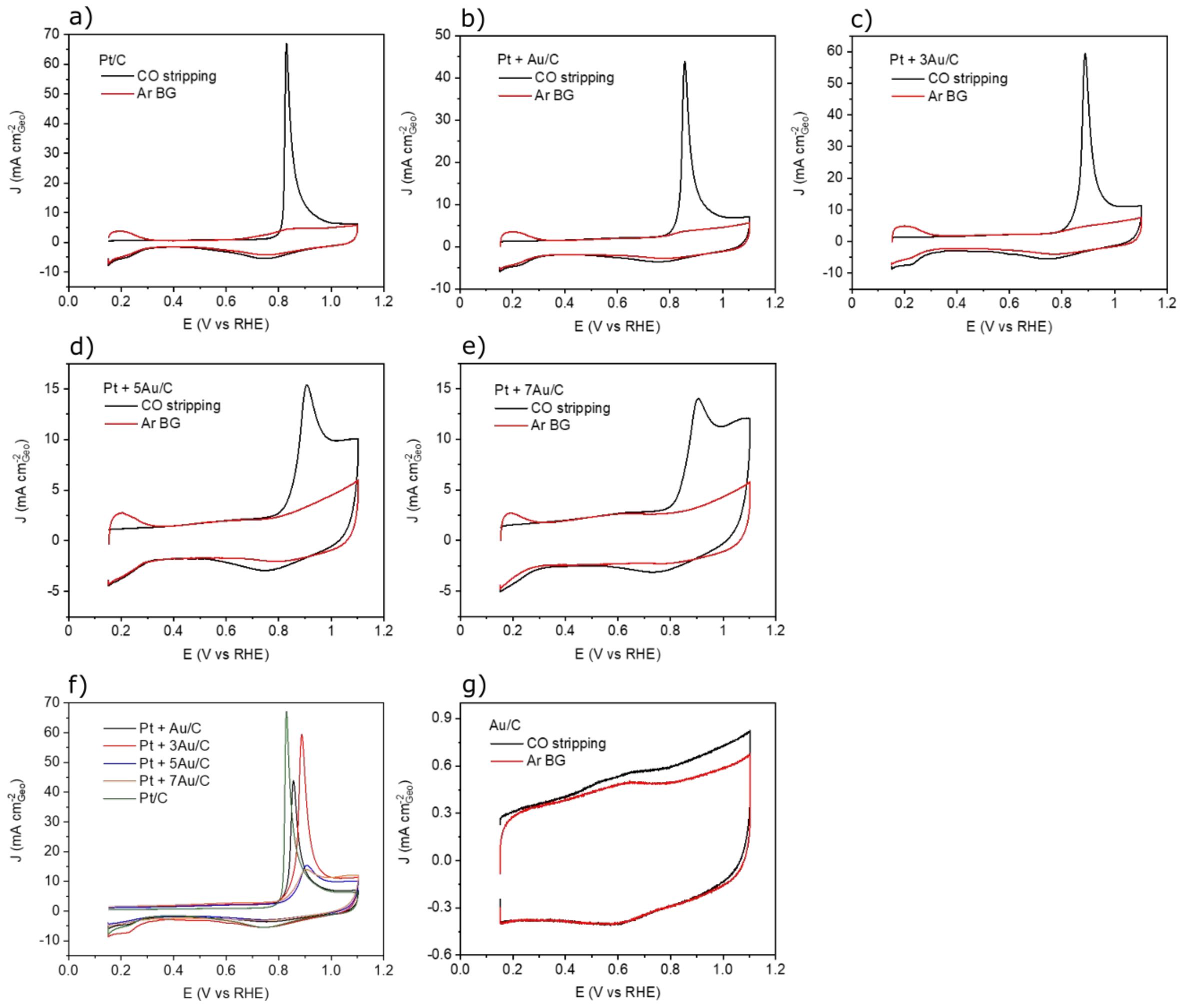

Figure S9. CO stripping curves and the subsequent CVs of the studied catalysts recorded in GDE measurements. The CO stripping curves of Pt-based catalysts are compared in Figure S9f. Scanning speed of 50 mV s^{-1} and 1.0 M $HClO_4$ serves as electrolyte in the upper cell body are applied for the measurements.

One can clearly observe the difference of CO stripping curves of the investigated catalysts. The catalysts with low Au content (one and three times higher of Au mass than Pt in composition) basically display the same feature as pure Pt/C, however, with Au loading increases to five (Figure S9d) and seven times (Figure S9e) higher than Pt, the CO oxidation peak seems to be incomplete in the standard potential window (0.15-1.10 V_{RHE}), therefore, an extended upper potential to 1.40 V_{RHE} is applied for these two catalysts and the CO stripping curves are exhibited in Figure S10, one can see a complete CO oxidation peak and thus only used to determine the ECSA of Pt +

5Au/C and Pt + 7Au/C. In addition, one can clearly observe that CO oxidation peaks are positively shifted with Au content (Figure S9f), as more Pt atoms are in direct contact with Au and thus the electronic modification is more pronounced. That is also the reason the standard potential window (0.15-1.10 V_{RHE}) is not sufficient for Pt + 5Au/C and Pt + 7Au/C in the CO stripping measurement.

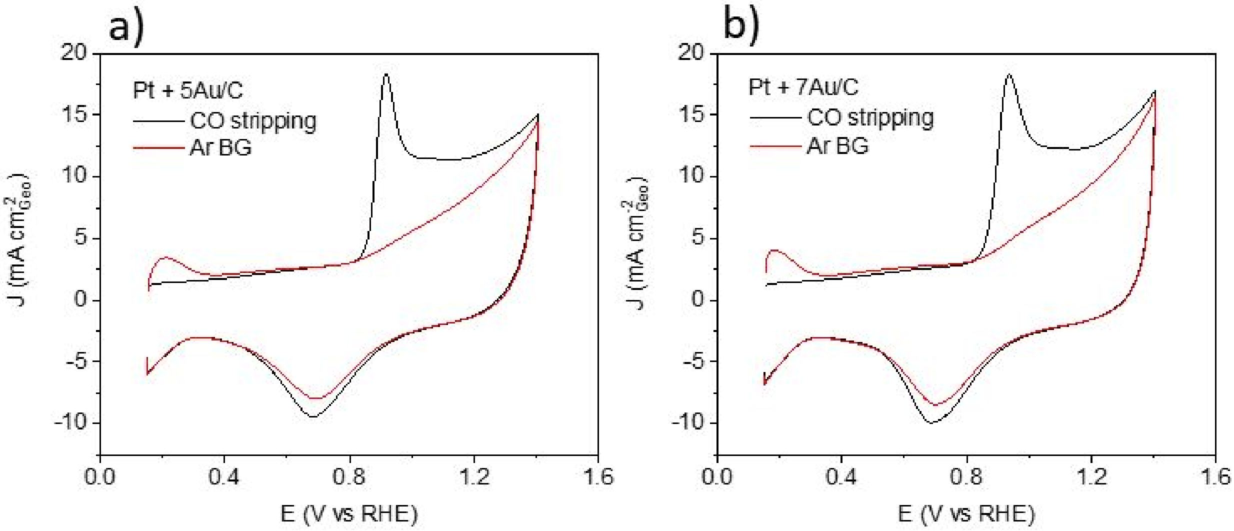

Figure S10. CO stripping curves and the subsequent CVs of the supported Pt + xAu/C nanocomposites (five and seven times higher than Pt composition in mass) with extended upper potential to 1.40 V_{RHE} in GDE setup. The scanning speed is 50 mV s^{-1} and the measurements are conducted with 1.0 M $HClO_4$ serves as electrolyte in the upper cell body in room temperature. The samples for the measurements are "one time" with no additional FAO measurements, to avoid pre-alloy of Pt and Au.

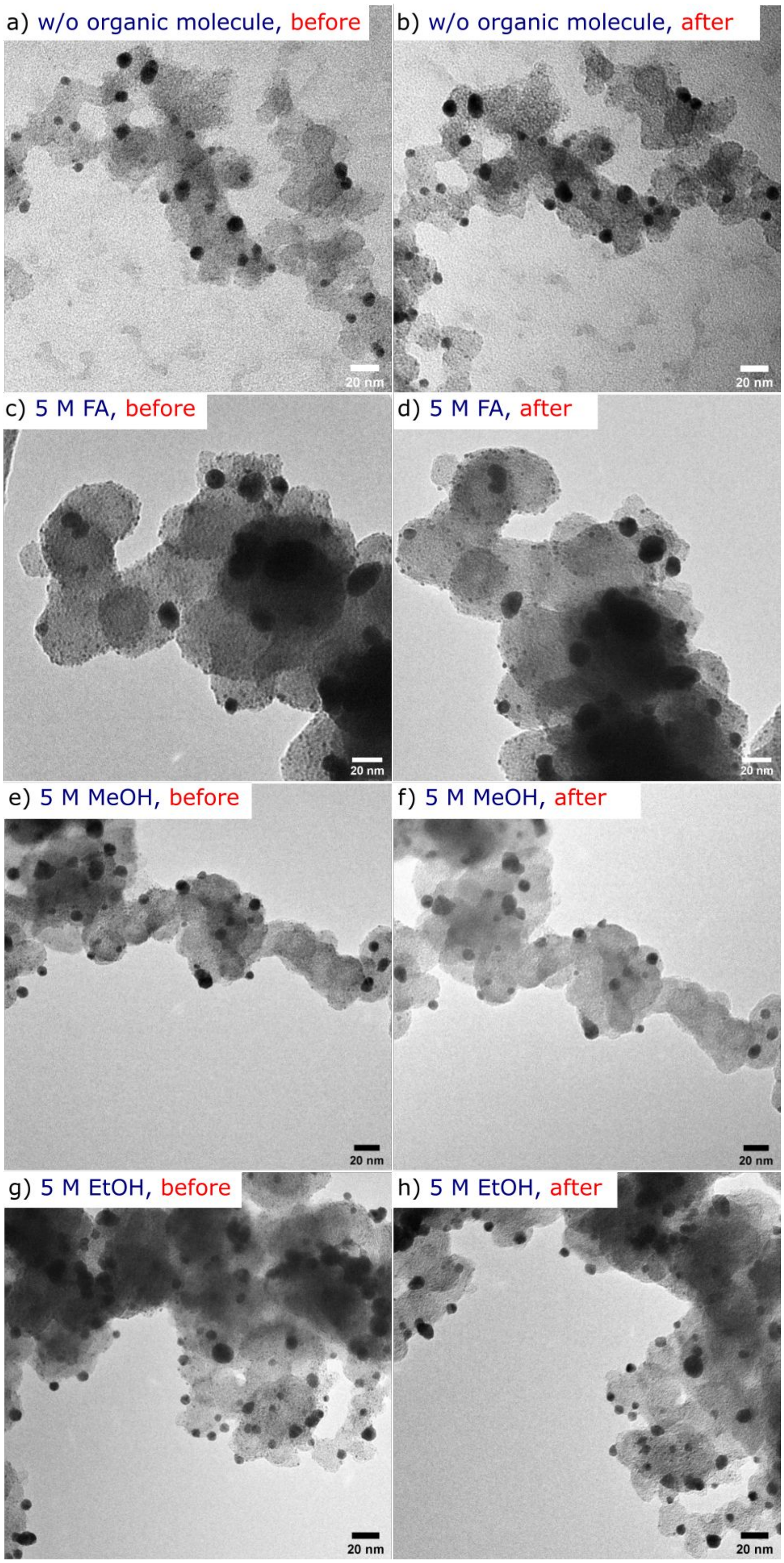
a) w/o organic molecule, before
b) w/o organic molecule, after
c) 5 M FA, before
d) 5 M FA, after
e) 5 M MeOH, before
f) 5 M MeOH, after
g) 5 M EtOH, before
h) 5 M EtOH, after
20 nm

Figure S11. IL-TEM of Pt + 5Au/C before (pristine sample) and after (after 50 CVs in the potentiodynamic test) electrochemical treatment in the GDE setup. The bubbler was filled with different organic solvents to change the reaction environment. A scan rate of 50 mV s^{-1} was applied for all measurements. The measurements demonstrate that without any organic molecules in the reaction environment, the metal particles (especially Pt particles) change the least upon exposition to the electrochemical treatment. By comparison, the Pt loss is more pronounced in an organic reaction environment. In MeOH or EtOH environment, Pt detachment seems to be more predominant leading to particle loss. However, from the measurements, it cannot be determined if *in situ* alloying occurs or not. In the FA environment, as already discussed in the manuscript, Pt dissolution and re-deposition occurs, leading to the formation of surface alloys (shown in the STEM-EDX measurements).

Table S5. The actual Pt loading (via ICP-MS) on GDL after vacuum filtration and the electrochemically Pt surface area (ECSA) of the investigated electrocatalysts. The ECSA values are normalized by the actual Pt mass on GDL. The upper potential in CO stripping measurements for ECSA determination is 1.10 V_{RHE} for Pt/C, Pt + Au/C and Pt + 3Au/C (Figure S9a-c), while an extended upper potential of 1.40 V_{RHE} is applied for Pt + 5Au/C and Pt + 7Au/C (Figure S10) to obtain a complete CO oxidation peak. Three independent CO stripping measurements are performed to add the standard deviations.

	Pt/C	Pt + Au/C	Pt + 3Au/C	Pt + 5Au/C	Pt + 7Au/C
Pt mass on GDL (μg)	8.8	5.6	8.3	8.3	7.1
ECSA (m^2 g_{Pt}^{-1})	110.8 ± 6.6	116.4 ± 8.6	123.0 ± 2.0	104.4 ± 5.8	104.8 ± 7.6

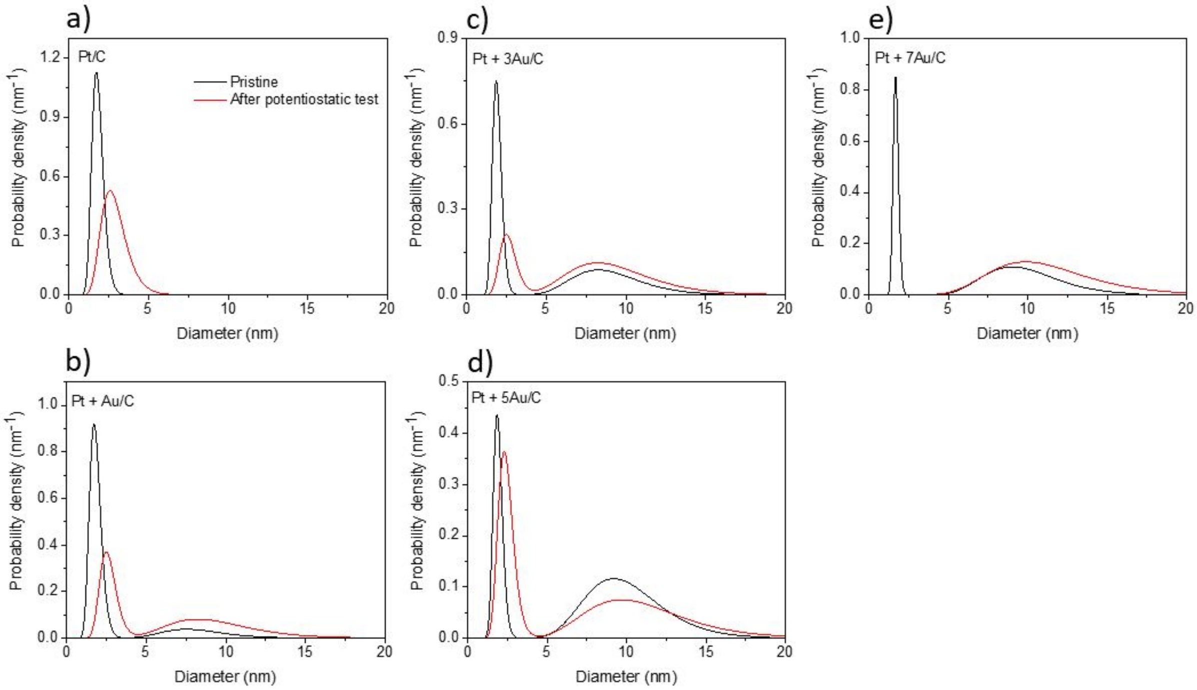

Figure S12. Size distribution comparison of the studied catalysts before and after potentiostatic test. All black curves represent the size distributions of each pristine sample, while all red curves are for the catalysts after potentiostatic test. The probability density of the particle size is derived from SAXS analysis with volume weighted.

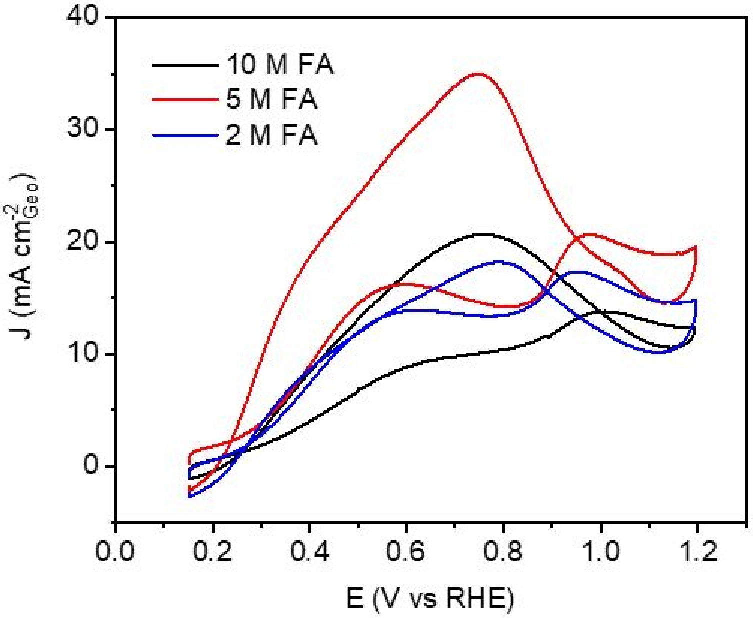

Figure S13. CVs of FAOR potentiodynamic test with different FA concentration in bubbler in GDE setup. The measurement is conducted on 2Pt + 4Au/C nanocomposite (the nominal Pt loading

is 20% in weight, all the rest nanocomposites in the study with a nominal Pt loading of 10% in weight). The upper cell body is filled up with 1.0 M $HClO_4$. A scanning speed of 50 mV s^{-1} is applied for all measurements.

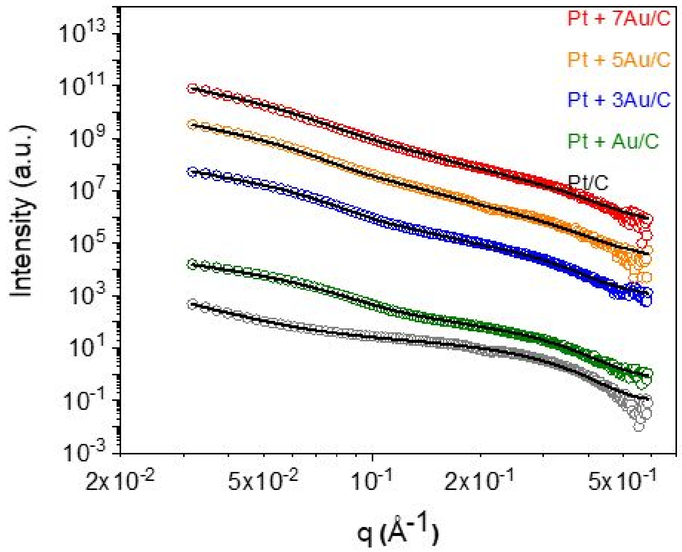

Figure S14. SAXS data and fits of the investigated pristine catalysts.

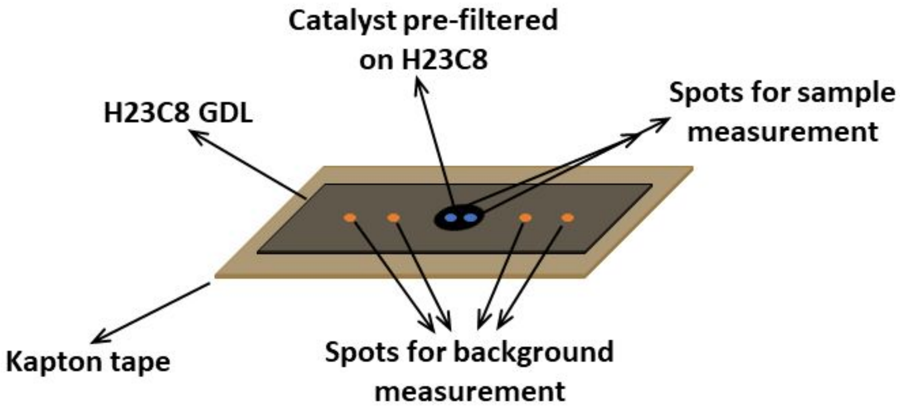

Figure S15. Sketch of WE inserted in a GDL stripe and fixed on the Kapton tape for *operando* SAXS measurements.
